# Development and Internal Validation of a Multivariable Prognostic Model for Sustained Remission of Depressive Symptoms in Middle-Aged and Older Adults: An Analysis of the CHARLS Cohort

**DOI:** 10.3390/healthcare14183112

**Published:** 2026-09-21

**Authors:** Alex Pak Lik Tsang, Patrick Pui Kin Kor

**Affiliations:** 1 Department of Sociology and Social Policy, Lingnan University, Tuen Mun, New Territories, Hong Kong SAR, China; 2 School of Nursing, The Hong Kong Polytechnic University, Hung Hom, Kowloon, Hong Kong SAR, China; patrick.kor@polyu.edu.hk

**Keywords:** depression, remission, prognostic model, machine learning, pain, older adults, CHARLS

## Abstract

**Background:** Depressive symptoms are common among middle-aged and older adults in China, and formal treatment is uncommon, so the course of symptoms often unfolds without intervention. Prognostic research has concentrated on who develops depression and who relapses after treatment, whereas remission from an already-symptomatic state has rarely been modeled. We developed and internally validated a model to predict sustained remission of depressive symptoms in a large community cohort. **Methods:** Using the China Health and Retirement Longitudinal Study (CHARLS), we identified adults aged 45 years and older with elevated depressive symptoms, defined as a score of 10 or higher on the 10-item Center for Epidemiologic Studies Depression Scale (CES-D-10), at a 2015 landmark. The outcome was sustained remission, defined as a score below 10 at both the 2018 and 2020 assessments. Fifty-four candidate predictors measured at or before the landmark were entered into a penalized logistic regression, with selection performed by the penalty within household-clustered cross-validation. Missing data were multiply imputed, discrimination was corrected for optimism using a household-cluster bootstrap, and geographic transportability was examined using internal–external validation. **Results:** Of 4117 participants, 818 (19.9%) achieved sustained remission. The model discriminated moderately, with an optimism-corrected C-statistic of 0.761, and was well calibrated, with a calibration slope of 1.06 and calibration-in-the-large of 0.00. Discrimination was carried principally by the recent symptom trajectory and, to a smaller degree, by pain. Behavioral, psychosocial, and sociodemographic predictors added little independent information. Performance was maintained when whole geographic regions were held out, with a pooled C-statistic of 0.754. A model restricted to information obtainable at a single assessment discriminated less well, with a C-statistic of 0.698, and discrimination was lower among participants with more persistent symptoms. **Conclusions:** Sustained remission was moderately and consistently predictable from routinely collected information, principally from the recent symptom trajectory and secondarily from pain. The findings support cautious prognostic stratification rather than individual prediction, with pain as a common and readily measured marker of reduced remission.

## 1. Introduction

Depressive symptoms in later life are associated with disability, greater health-care use and costs, and elevated mortality [1,2]. In China, meta-analyses have estimated that approximately one fifth to one quarter of older adults experience clinically relevant depressive symptoms [3,4]. However, people with depressive disorders frequently receive no formal care. In the nationally representative China Mental Health Survey, only 9.5% of people with a 12-month depressive disorder received treatment from any health-service institution, and only 0.5% received adequate treatment [5]. Understanding who does and does not improve under naturalistic conditions is therefore relevant to the monitoring and prioritization of limited mental-health services.

The course of depressive symptoms in later life is heterogeneous. In a large longitudinal cohort of older adults, Agustini, Lotfaliany [6] identified consistently low, moderate, emerging, and persistently high symptom trajectories, illustrating that symptom burden does not follow a uniform course. The prognostic literature has addressed primarily who will develop depression [7,8], who will relapse or experience recurrence after remission [9], and who will respond to treatment [10]. Prediction of sustained remission among people who are already symptomatic, particularly in community populations not selected for treatment, remains comparatively underdeveloped.

Prediction studies using Chinese longitudinal cohorts have likewise focused predominantly on symptom onset. Zheng, Zhang [11] developed models for incident two-year depression among older adults with chronic diseases using the 2011 to 2015 CHARLS waves and conducted temporal validation using 2018 to 2020 data. Their selected model showed modest discrimination in temporal validation, with an area under the receiver operating characteristic curve of 0.623, and generally satisfactory calibration. Instrumental activities of daily living disability appeared among the higher-ranked features in several algorithms. Huang, Jiang [7] similarly excluded participants with depressive symptoms at baseline and applied machine-learning methods to predict subsequent symptom onset. Such models address who is likely to become symptomatic, which is important for prevention but differs from estimating prognosis once a symptomatic state has been established. Risk factors for developing a condition and prognostic factors governing its subsequent course are conceptually distinct and need not have the same predictive importance [12]. Although mental-health resources have expanded in China, national evidence indicates substantial geographical inequity in the distribution of psychiatric beds and persistent disparities in the availability and training of mental-health professionals between eastern urban areas and western or rural areas [13,14]. In this context, a transparent model estimating who is less likely to remit without intervention could inform decisions about closer monitoring and the prioritization of limited clinical support.

Several considerations informed the candidate predictors of sustained remission from depressive symptoms. First, recent symptom trajectory is a strong candidate because residual symptoms, symptom severity, and previous depressive history are consistently associated with relapse and recurrence [15,16], and repeated population assessments allow this history to be represented directly. Second, pain is a plausible physical-health predictor because pain and depression commonly co-occur and appear to have reciprocal longitudinal relationships in aging populations [17,18]. Third, physical activity, socioeconomic circumstances, and social relationships have been associated with prevalent or incident depression [19,20,21], although their independent contribution to remission conditional on already being symptomatic is less certain. Finally, if prognosis can be estimated from routinely collected information, risk stratification could help identify people who may require more intensive monitoring or support, consistent with stepped and stratified approaches to care [22,23,24]. The intended users of such a model are clinicians, primary-care and community health workers, and service planners.

Penalized regression permits a broad, prespecified candidate set to be entered together, with selection and shrinkage performed inside internal validation, which reduces the optimism that follows from data-driven predictor screening [25,26]. We therefore developed and internally validated a multivariable prognostic model to predict sustained remission of depressive symptoms among middle-aged and older adults in CHARLS. Our objectives were to quantify how well sustained remission could be predicted from information available at or before a landmark assessment, to determine which predictor domains carried independent prognostic information, and to examine calibration and geographic transportability across regions within China.

## 2. Method

### 2.1. Study Design and Data Source

This study developed and internally validated a multivariable prognostic model for sustained remission of depressive symptoms among middle-aged and older adults in a community cohort. The study was informed by the PROGnosis RESearch Strategy (PROGRESS) framework for prognostic research [26,27] and is reported with reference to the TRIPOD+AI statement [28]. Geographic transportability was additionally examined using internal–external validation, in which geographically defined groups were omitted sequentially from model development and used for validation [29,30].

Data were obtained from the China Health and Retirement Longitudinal Study (CHARLS). CHARLS used a multistage probability-sampling design to recruit community-dwelling adults aged 45 years or older and their spouses from 28 provinces in China, and the baseline cohort was designed to be nationally representative of this population [31]. The present study used information collected in the 2011, 2013, 2015, 2018, and 2020 waves.

### 2.2. Participants, Prediction Origin, and Eligibility

The prediction landmark, the time origin from which prognosis was estimated, was the 2015 assessment. Participants were eligible if they had elevated depressive symptoms at the landmark, operationalized as a score of 10 or higher on the 10-item Center for Epidemiologic Studies Depression Scale (CES-D-10). The CES-D-10 was developed as a brief screening instrument, and a cutoff of 10 has been recommended for identifying elevated symptoms among older adults [32]. This threshold indicates screening-level symptom elevation and should not be interpreted as a clinical diagnosis of depressive disorder.

A total of 6356 participants met the symptom criterion at the landmark. Of these, 4117 completed both follow-up assessments required to determine the primary outcome and constituted the primary analytic sample. All predictors were measured at or before the 2015 landmark. The outcome was assessed three and five years after the landmark, in 2018 and 2020, respectively.

### 2.3. Outcome

The primary outcome was sustained remission of depressive symptoms, operationalized as a CES-D-10 score below 10 at both the 2018 and 2020 assessments. In this study, *remission* refers to symptom scores below the prespecified screening threshold and does not denote clinician-established remission from a depressive disorder, while *sustained* indicates that the threshold criterion was met at both follow-up assessments. Because symptoms were not observed continuously between waves, continuous remission throughout the interval cannot be established. This operational definition incorporates movement into a range below the screening threshold, corresponding to the nonclinical-range component of the clinical-significance framework [33]. It does not apply a reliable-change criterion and should be regarded as a study-specific definition. The term *recovery* was avoided as it implies more sustained resolution than can be established from two widely separated symptom assessments [9].

A sensitivity outcome, pre-pandemic remission, was defined as a CES-D-10 score below 10 at the 2018 assessment. This analysis examined whether predictability was already evident before the 2020 assessment. Because death precludes subsequent assessment of remission and differs from ordinary missing follow-up, a further sensitivity analysis redefined the favorable outcome as being alive and in sustained remission [34,35]. Participants known to have died before the final assessment were therefore classified as not achieving this composite outcome. This does not assume that deceased participants remained symptomatic. It examines whether conditioning the primary analysis on survival influenced model performance. Deaths were identified through the 2018 interview-status records, so deaths occurring between 2018 and 2020 were not captured, and the analysis represents a lower-bound assessment of mortality-related selection.

### 2.4. Candidate Predictors

Candidate predictors were specified before model fitting on the basis of prior literature concerning depression onset and course. The complete candidate set comprised 54 variables organized into five conceptual blocks.

The illness-course block included CES-D-10 severity at the 2015 landmark and the 2013 and 2011 waves, prior-wave elevated-symptom status, change in symptoms between 2013 and 2015, number of previous waves with elevated symptoms, and somatic and affective symptom-subscale scores. Symptom severity, residual symptoms, and prior depressive history have been consistently associated with subsequent depressive course [15,16].

The physical-health block included number of chronic conditions, activities of daily living and instrumental activities of daily living limitations, gross-motor mobility limitation, body mass index, global cognition, presence and number of sites of bodily pain, distance and near vision, hearing problems, falls, childhood health, and lagged self-rated health. Pain and depression frequently co-occur, and reciprocal longitudinal associations have been reported in aging cohorts [17,18].

The lifestyle block included smoking history and current smoking, alcohol consumption and frequency, vigorous, moderate, and light physical activity, and sleep duration.

The psychosocial block included social activities, marital status, widowhood transition, living arrangement, number of living children and siblings, and intergenerational financial transfers.

The sociodemographic block included age, sex, education, household registration, household wealth, and wage income. These domains were included because physical activity, social relationships, and socioeconomic circumstances have been associated with prevalent or incident depression, although their prognostic importance after symptom elevation is established remains less certain [19,20,21]. Detailed definitions and coding are presented in Table S1, and the rationale for each block is summarized in Table S2.

Continuous, count, ordinal, and binary predictors were standardized to a mean of zero and standard deviation of one so that the elastic-net penalty operated on a common scale. Count and ordinal variables were entered as single linear terms. Marital status and household registration were represented as partnered versus unpartnered and rural versus urban, respectively. Concurrent self-rated health and life satisfaction were excluded because they may partly reflect the index mood state. Self-rated health was instead entered using its 2013 value to ensure that it preceded the prediction landmark. The CES-D restless-sleep item was not entered as a separate sleep predictor because it was already represented within the CES-D total score, and sleep behavior was represented separately by reported sleep duration. Anxiety was not measured in CHARLS and could not be included, although it has been associated with an adverse depressive course [15]. Informal care from children was excluded because 73% of values were missing, and detailed income and treatment variables were excluded because of insufficient coverage. Household wealth and transfer amounts were transformed using a signed logarithm to reduce the influence of extreme values.

### 2.5. Sample Size

Because the study used all eligible participants from an existing cohort, the available sample size was fixed. The primary sample provided 818 events for 54 candidate parameters, approximately 15 events per parameter, which exceeds the conventional minimum of about 10 [36]. Adequacy was assessed using the criteria from Riley, Snell [37] and Riley, Ensor [38], considering all 54 candidate parameters rather than only predictors retained after penalization. With an outcome prevalence of 0.199, the required sample was approximately 3260 under a scenario based on the optimism-corrected C-statistic of 0.76 and approximately 5700 under a more conservative C-statistic of 0.70. The available sample of 4117 participants therefore lay between these benchmarks.

The calculation based on observed performance is post hoc and was used for contextual assessment rather than as prospective justification. To limit overfitting, all candidate predictors were retained for penalized estimation, and model performance was evaluated using household-clustered cross-validation and bootstrap optimism correction. Elastic-net penalization shrinks coefficients and reduces the effective number of parameters, which helps to limit overfitting [25]. The estimated optimism and calibration slope reported below provide direct empirical evidence on this point.

### 2.6. Missing Data

Missing predictor values were handled using multiple imputation by chained equations under a missing-at-random assumption [39,40]. Continuous variables were imputed using predictive mean matching and binary variables using logistic regression. The imputation model included the outcome and all candidate predictors. Including the outcome improves imputation of missing predictor values during model development, and the imputation model should contain the variables included in the analysis model [40,41].

Thirty imputed datasets were generated using five iterations of the chained-equations algorithm, and convergence was assessed by inspecting iteration trace plots of the mean and standard deviation of imputed values against iteration number (Supplementary Figure S1). Unpenalized estimates requiring standard errors were combined using standard multiple-imputation rules [42]. Penalized coefficients and prediction-performance measures were calculated within each completed dataset and averaged across the 30 datasets. Because conventional Rubin variance estimates are not directly defined for penalized coefficients, confidence intervals were not computed for those coefficients.

Imputation was conducted at the individual level without a multilevel random-effects structure. Household dependence was accommodated during validation by retaining households intact in cross-validation and resampling complete households in the bootstrap. Imputation was completed before cross-validation and bootstrap resampling and was not repeated within each resampling iteration. The reported validation estimates are therefore conditional on the completed datasets and do not represent validation of the entire imputation and model-development pipeline. The 2013 and 2011 CES-D-10 scores were missing for a substantial proportion of participants, predominantly because refreshment-sample members had not participated in the earlier waves. We quantified the source of this missingness and repeated model development among participants with complete prior-wave symptom data.

### 2.7. Model Development

Analyses were conducted in R (version 4.3.2). The primary model was a penalized logistic regression fitted using elastic net [25,43]. The mixing parameter was fixed a priori at 0.50 to retain a balance between coefficient shrinkage and variable selection. The penalty parameter was selected using household-clustered five-fold cross-validation. Within each completed dataset, households were allocated intact to folds so that members of the same household, including married couples, were never divided between training and validation partitions. The complete candidate set was entered simultaneously, and no univariable significance screening was used to determine model eligibility. This approach is consistent with recommendations to prespecify candidate predictors and use shrinkage rather than preliminary significance testing for model selection [26]. The penalty was selected within each imputed dataset, and out-of-fold predicted probabilities were used to estimate cross-validated performance. Coefficients and performance measures were subsequently averaged across the 30 completed datasets. In restricted sensitivity samples, any predictor that became invariant was excluded from that model fit, and small amounts of residual missingness arising in restricted analyses were replaced by the corresponding subset median before estimation.

### 2.8. Internal Validation and Model Performance

Discrimination was summarized using the C-statistic. Apparent performance was corrected for optimism using 500 household-cluster bootstrap resamples [44]. In each resample, the model-development procedure was repeated, and the resulting model was evaluated in the bootstrap sample and the original completed dataset. Optimism was estimated as the average difference between these two performance estimates. The principal cross-validated C-statistic was calculated from the out-of-fold predictions. Its 95% confidence interval was obtained by resampling households rather than from variability among the five cross-validation folds. Because imputation was not repeated during resampling, the cross-validated and bootstrap estimates are conditional on the previously completed datasets. Calibration was evaluated using the calibration slope, calibration-in-the-large, and a smoothed calibration plot accompanied by the distribution of predicted probabilities [45]. Overall prediction error was summarized using the Brier score. Potential clinical utility was explored using decision-curve analysis [46]. Because the fitted model estimated the probability of remission, predicted probabilities were complemented to obtain the probability of non-remission. Net benefit was examined for decisions to flag participants at elevated predicted probability of non-remission across threshold probabilities from 0.30 to 0.90. These analyses are exploratory and do not establish that model-guided decisions improve clinical outcomes. The complete averaged model specification, including the intercept, standardized coefficients, development-sample means, and standard deviations, is reported in Table S3.

### 2.9. Internal–External Validation

Geographic transportability was assessed using internal–external validation [29,30]. The 27 provinces represented in the analytic sample were grouped into five folds. Province was selected as the grouping unit because different regions may differ in population composition and service context. The 27 provinces were allocated at random to the five folds using a fixed random seed, so the folds are groups of provinces rather than administratively or economically defined regions. The provinces assigned to each fold, with the corresponding sample sizes and event counts, are reported in Table S6. The model was developed using four folds and evaluated in the omitted fold, and this process was repeated until every fold had served as the validation sample. Each estimate was therefore produced by a model that had not been trained using participants from the provinces in that fold. Held-out C-statistics were summarized across folds, and the range of estimates was examined descriptively. This procedure evaluates transportability to provinces not represented in model development but does not constitute external validation in an independent cohort. As in the primary analysis, the internal–external validation was performed within the previously completed imputed datasets, and imputation was not repeated when folds were held out. The held-out estimates are therefore conditional on those datasets and do not evaluate the imputation and model-development pipeline as a whole.

### 2.10. Sensitivity and Stability Analyses

Several sensitivity analyses were conducted. First, the model was fitted to remission at the 2018 wave to examine performance before the 2020 assessment. Second, performance was compared with that of a gradient-boosting model [47]. Third, the complete model was compared with a parsimonious model restricted to illness-course and pain predictors. Fourth, we examined whether using the CES-D-10 for both eligibility and outcome definition inflated the apparent contribution of symptom severity. Analyses were repeated in three restricted samples: participants with landmark scores of at least 12, participants with landmark scores of at least 15, and participants with elevated symptoms at both the 2013 and 2015 waves. Fifth, we evaluated a single-contact model restricted to information potentially obtainable during one assessment, comprising the 2015 CES-D-10 score, pain presence, number of pain sites, age, sex, education, and household registration. Sixth, attrition and death were examined by comparing participants included in and excluded from the primary analysis, redefining the favorable outcome as being alive and in sustained remission, and conducting an extreme sensitivity analysis in which all excluded participants were classified as non-remitters. The latter analysis depends on the unverifiable assumption that no excluded participant achieved remission and was used only to assess sensitivity. The unique predictive contribution of the illness course block was evaluated by removing that block from the model. Selection stability was examined by recording the proportion of 500 household-bootstrap samples in which each predictor received a nonzero coefficient. Selection frequency was interpreted as a measure of stability under resampling rather than as a measure of causal importance or effect size.

## 3. Results

### 3.1. Participant Flow and Sample Characteristics

**Figure 1:**
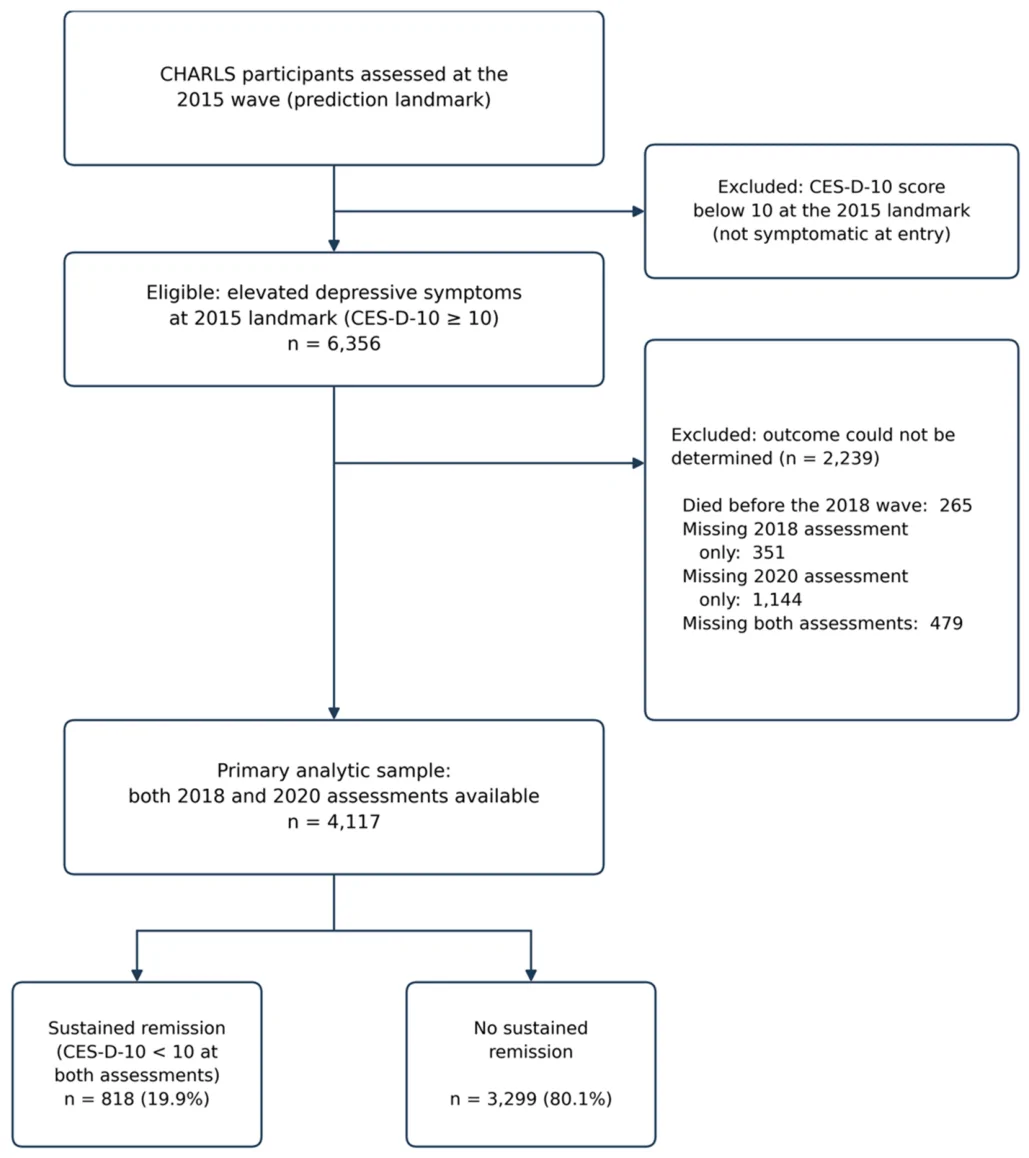
Flow of Participants Through the Study. Note. Sustained remission was defined as a 10-item Center for Epidemiologic Studies Depression Scale (CES-D-10) score below 10 at both the 2018 and 2020 assessments. Deaths were identified from the 2018 interview-status records; deaths occurring between the 2018 and 2020 assessments were not captured, so the number who died before the final assessment is a lower bound. Percentages are of the primary analytic sample.

Among 6356 participants with elevated depressive symptoms at the 2015 landmark, 2239 lacked one or both assessments required to define the primary outcome. Of these, 265 were known to have died before the 2018 wave, 351 lacked the 2018 assessment only, 1144 lacked the 2020 assessment only, and 479 lacked both. The primary analytic sample therefore comprised 4117 participants, of whom 818 (19.9%) met the definition of sustained remission (Figure 1).

Excluded participants were older than included participants (mean 62.9 years, SD 11.2, versus 57.9 years, SD 9.2). They also had greater instrumental activities of daily living limitations (0.97 versus 0.61), activities of daily living limitations (0.82 versus 0.57), and mobility limitations (2.64 versus 2.13). Differences in landmark depressive symptoms (15.7 versus 15.2) and pain prevalence (56.2% versus 54.8%) were smaller. Attrition was therefore more strongly patterned by age and functional impairment than by landmark depressive-symptom severity or pain, although selection related to unmeasured characteristics cannot be excluded (Table S4).

Characteristics of the analytic sample by outcome are presented in Table 1. Compared with non-remitters, participants who achieved sustained remission had lower CES-D-10 scores at the landmark and both previous waves, fewer prior waves with elevated symptoms, lower levels of comorbidity and functional impairment, and less pain. In 2013, 33.4% of remitters and 63.8% of non-remitters with available data had elevated symptoms. Pain was reported by 37.7% of remitters and 59.0% of non-remitters, and the corresponding mean numbers of pain sites were 1.74 and 3.43. Remitters also had modestly higher cognitive scores.

### 3.2. Sample-Size Assessment and Estimated Optimism

The apparent C-statistic was 0.774, and estimated bootstrap optimism was 0.012, yielding an optimism-corrected C-statistic of 0.761. The calibration slope was 1.06, above the shrinkage target of 0.90 that the sample-size criteria are designed to maintain. These findings indicate that the model was estimated without material overfitting in the completed datasets. Because imputation was not repeated within resampling, some residual optimism arising from preprocessing cannot be excluded.

### 3.3. Predictor Associations

In household-clustered univariable analyses, prior depressive-symptom measures had the strongest associations with sustained remission, followed by the pain measures (Table 2). Functional impairment, comorbidity, poorer sensory functioning, falls, widowhood transition, female sex, and rural household registration were associated with lower odds of remission, whereas cognition was associated with higher odds. These analyses were descriptive and did not determine entry into the multivariable model.

**Figure 2:**
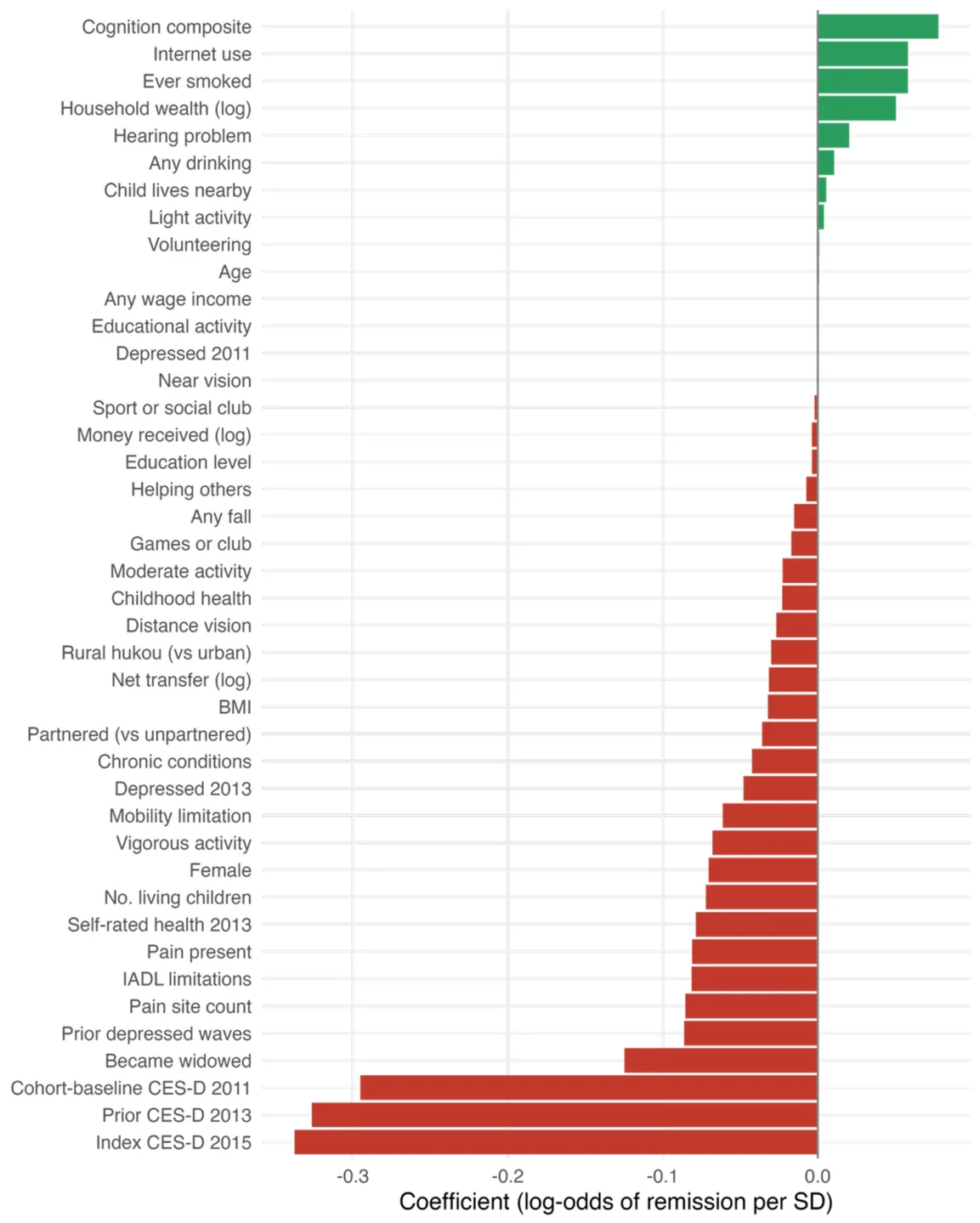
Standardized Coefficients From the Penalized Multivariable Model. Note. Bars show elastic-net coefficients on the standardized scale, averaged across 30 imputed datasets, expressed as the change in the log-odds of sustained remission per standard deviation of the predictor. Negative values indicate lower odds of remission. Predictors reduced exactly to zero at the selected penalty are not displayed. Coefficients are conditional prediction weights. The complete specification, including the intercept and standardization constants, is given in Table S3.

In the penalized multivariable model, CES-D-10 severity in 2015, 2013, and 2011 received the largest coefficients. Smaller nonzero coefficients were retained for widowhood transition, number of previous waves with elevated symptoms, number of pain sites, instrumental activities of daily living limitations, and pain presence (Figure 2). Most behavioral, psychosocial, and sociodemographic coefficients were either reduced to zero or assigned small weights after simultaneous adjustment.

Ever smoking, alcohol consumption, internet use, and household wealth received small positive coefficients. However, these estimates should not be interpreted as evidence of protective causal effects, because penalized coefficients are conditional prediction weights and may reflect confounding, correlation among predictors, or sampling variability.

Table 2 reports elastic-net coefficients averaged across the 30 completed datasets and exponentiated as odds ratios. Conventional confidence intervals were not calculated for these penalized estimates. A coefficient of exactly zero indicates that the predictor was excluded at the selected penalty conditional on the other included predictors, and does not demonstrate the absence of an unadjusted association with remission.

### 3.4. Model Performance

**Figure 3:**
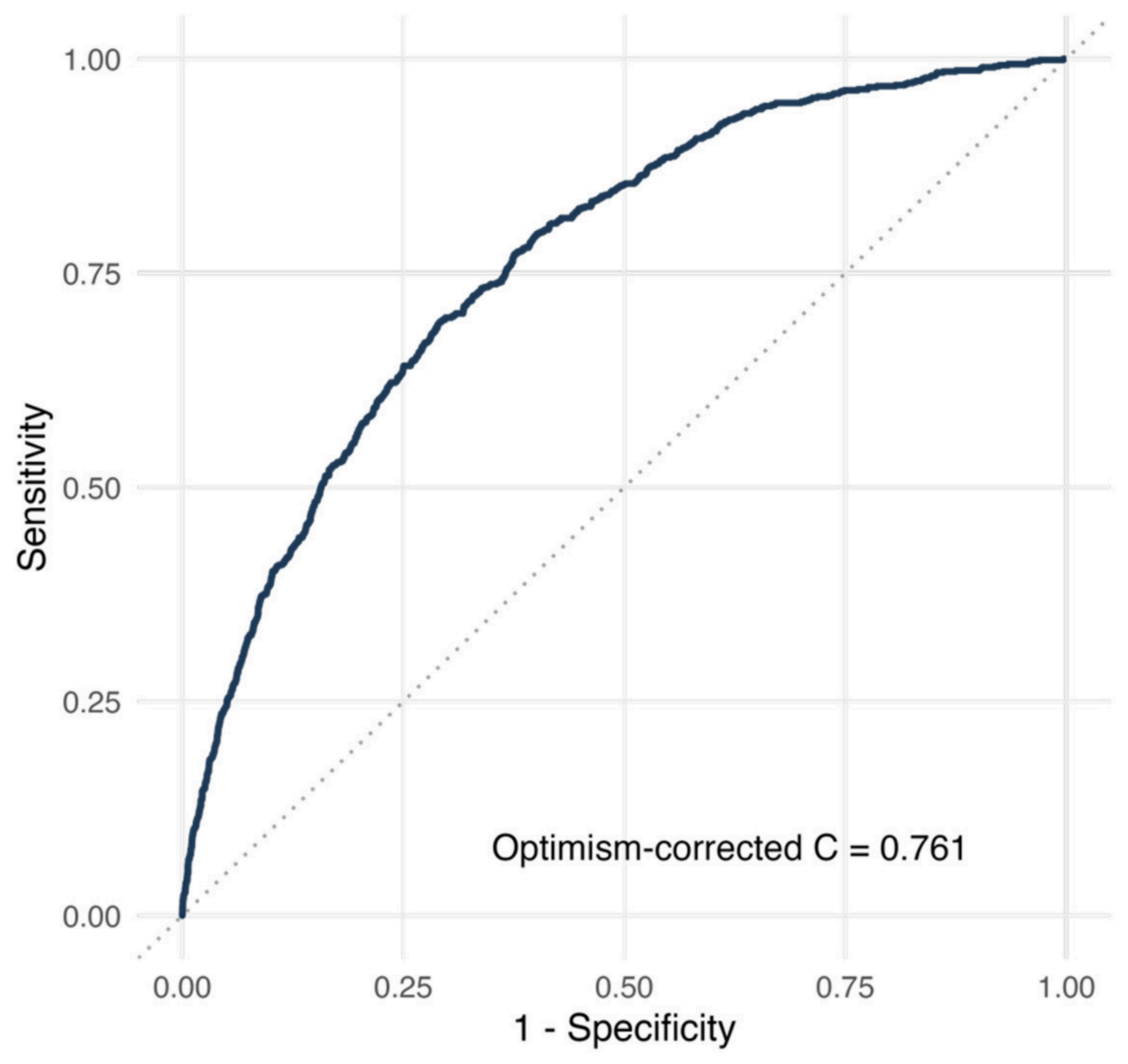
Receiver Operating Characteristic Curve for the Full-Candidate Model. Note. The curve is based on pooled out-of-fold predicted probabilities from household-clustered five-fold cross-validation, averaged across 30 imputed datasets. The optimism-corrected C-statistic shown was obtained from 500 household-cluster bootstrap resamples. The diagonal reference line indicates the performance expected by chance.

**Figure 4:**
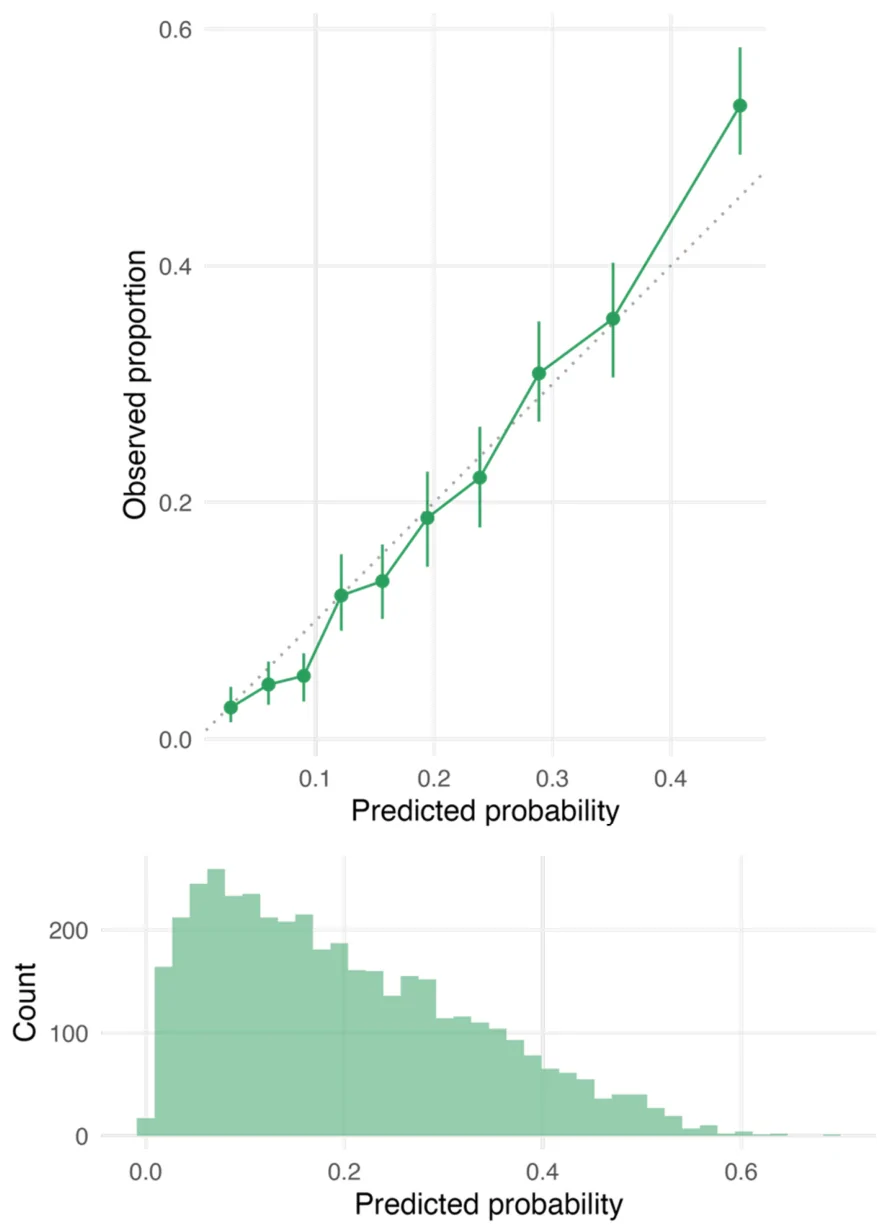
Calibration Plot for Predicted Probability of Sustained Remission. *Note.* Points show the observed proportion achieving sustained remission against mean predicted probability within deciles of predicted risk. Vertical bars are 95% confidence intervals obtained by resampling households. The dotted line indicates perfect calibration. The accompanying histogram shows the distribution of predicted probabilities across the analytic sample; predicted probabilities were concentrated below 0.40, so calibration in the upper range is estimated from relatively few participants.

The model showed moderate discrimination and close agreement between predicted and observed outcome frequencies (Table 3). The optimism-corrected C-statistic was 0.761, and the pooled cross-validated C-statistic was 0.755 (95% CI [0.749, 0.784]). Calibration-in-the-large was 0.00, indicating that mean predicted and observed outcome frequencies were aligned. The calibration slope was 1.06, close to the ideal value of 1, indicating that predictions were slightly insufficiently dispersed rather than excessively extreme. The Brier score was 0.138. The receiver operating characteristic curve and smoothed calibration plot are presented in Figure 3 and Figure 4.

**Figure 5:**
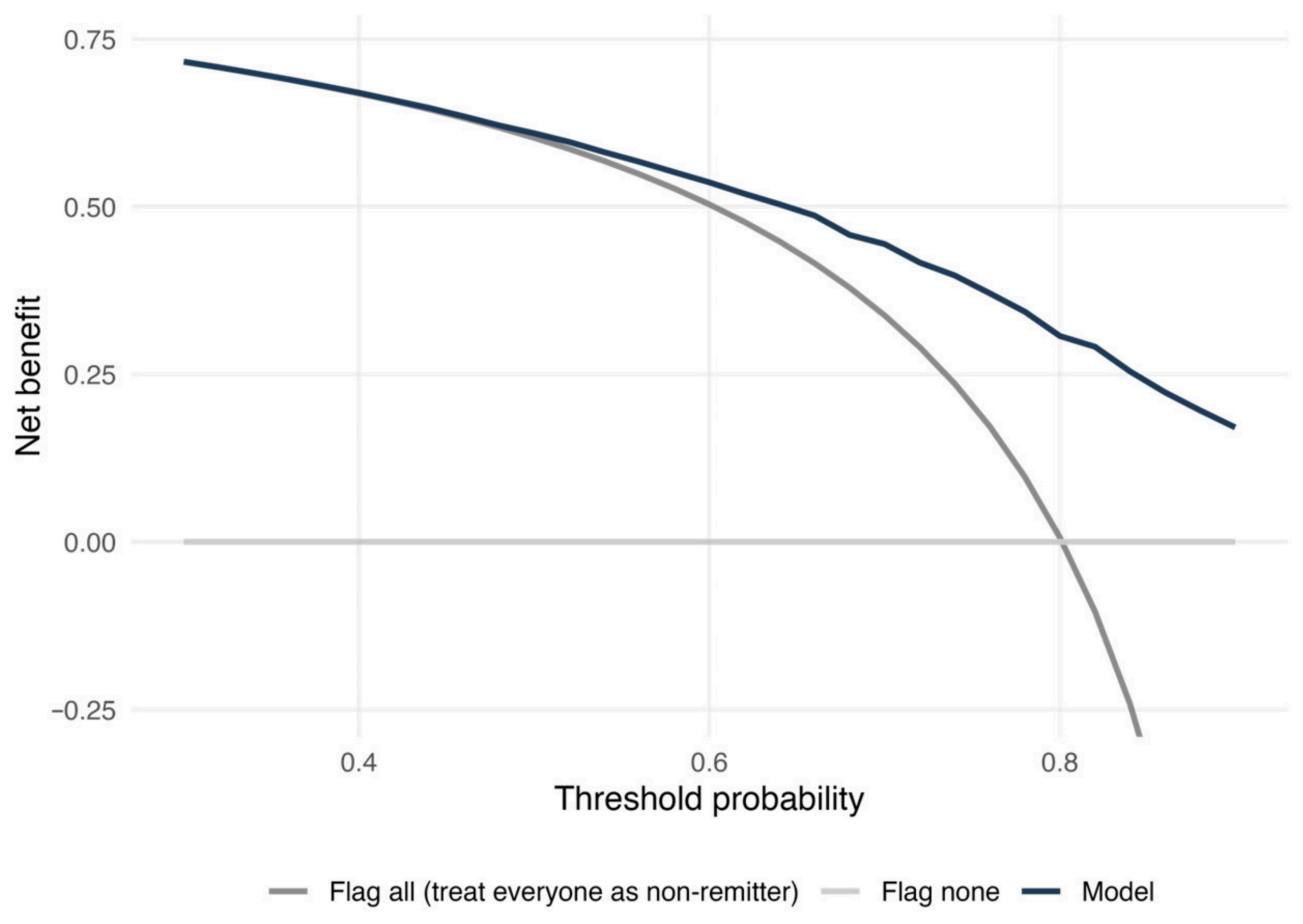
Decision-Curve Analysis for Identifying Participants Unlikely to Remit. *Note.* Net benefit is plotted against threshold probability for three strategies: using the model, flagging all participants as unlikely to remit, and flagging none. Because the model estimates the probability of remission, predicted probabilities were complemented to obtain the probability of non-remission. The threshold probability represents the predicted probability of non-remission at which a clinician would consider additional monitoring or support warranted.

Decision-curve analysis indicated that, within the examined threshold range, use of predicted non-remission probabilities produced greater estimated net benefit than flagging all participants, with a larger difference above a threshold of approximately 0.50 (Figure 5). This analysis indicates potential decision value under the specified assumptions but does not establish clinical effectiveness.

### 3.5. Predictor-Block Contribution and Selection Stability

**Figure 6:**
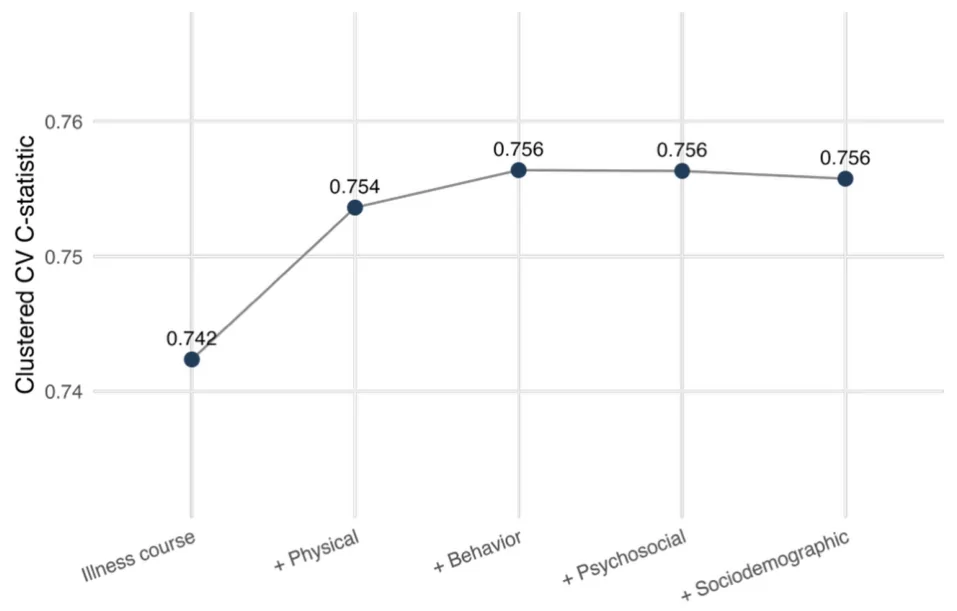
Discrimination by Sequential Addition of Predictor Blocks. *Note.* Values are household-clustered cross-validated C-statistics for models containing the illness-course block alone and then with each subsequent block added. Because blocks were added in a fixed order, the incremental contribution attributed to each later block depends on that ordering.

Most discrimination was obtained from the illness-course predictors. The illness-course block alone produced a C-statistic of 0.742. Adding physical-health predictors increased the C-statistic to 0.754. Adding the lifestyle, psychosocial, and sociodemographic blocks produced no material further improvement, with estimates of approximately 0.756 after rounding (Figure 6). A parsimonious model containing illness-course and pain predictors produced a C-statistic of 0.755 and a calibration slope of 1.02, closely matching the full model.

Removing the illness-course block reduced the C-statistic to 0.695. This comparison indicates that recent and previous depressive-symptom measures contained predictive information that was not recovered by the remaining candidate variables.

The three CES-D-10 scores were retained in all 500 bootstrap samples. Widowhood transition was retained in 99.8%, vigorous physical activity in 98.8%, cognition and lagged self-rated health in 97.6%, instrumental activities of daily living limitations in 95.6%, pain presence in 92.6%, and pain-site count in 89.8% (Table S5). High inclusion frequency indicates stability of a nonzero coefficient under the fitted penalization procedure and does not necessarily imply a large coefficient or meaningful incremental improvement in overall prediction.

The gradient-boosting comparator produced a C-statistic of 0.735 and a calibration slope of 0.78. It therefore did not improve discrimination and produced predictions that were more extreme than supported by the observed outcomes.

### 3.6. Sensitivity Analyses

Sensitivity analyses are summarized in Table 4. For remission at the 2018 wave, the C-statistic was 0.715. Predictive information was therefore evident before the 2020 assessment, although discrimination was lower than for the primary repeated-wave outcome.

Restricting eligibility to participants with a landmark CES-D-10 score of at least 12 produced a C-statistic of 0.754. The C-statistic declined to 0.721 when eligibility required a score of at least 15. Among participants with elevated symptoms in both 2013 and 2015, the C-statistic was 0.706, and the full and illness-course-only models produced similar values of 0.706 and 0.704, respectively. Discrimination therefore attenuated when the sample was restricted to participants with more severe or persistent symptoms but remained moderate.

The single-contact model produced a C-statistic of 0.698 and a calibration slope of 1.01. Information from previous symptom assessments therefore provided appreciable additional discrimination beyond information obtainable from one assessment.

Among missing 2013 and 2011 CES-D-10 scores, 83% and 86%, respectively, occurred because participants had not been surveyed in those waves. In the 2645 participants with complete prior-wave symptom data, the C-statistic was 0.764, and the calibration slope was 1.09. Discrimination was therefore not lower in the subset for whom prior-wave values were observed rather than imputed, which does not support the concern that imputation generated the apparent prognostic signal. This subset nevertheless differed from the complete analytic sample, and the comparison does not verify the missing-at-random assumption.

When the favorable outcome was redefined as being alive and in sustained remission, with known deaths classified as not achieving that outcome, the remission proportion decreased from 19.9% to 18.7%, whereas discrimination remained essentially unchanged (C-statistic 0.759 versus 0.755 in the primary analysis). Exclusion of the recorded deaths therefore did not materially account for the model’s discrimination. Because deaths occurring between 2018 and 2020 were not comprehensively captured, the analysis does not quantify the influence of all mortality before the final assessment.

In the extreme analysis classifying every excluded participant as a non-remitter, the full model produced a C-statistic of 0.750 and the single-contact model a C-statistic of 0.704. These results indicate numerical robustness to the imposed classification, although the analysis relies on the implausible assumption that no surviving non-responder achieved remission.

### 3.7. Internal–External Validation

**Figure 7:**
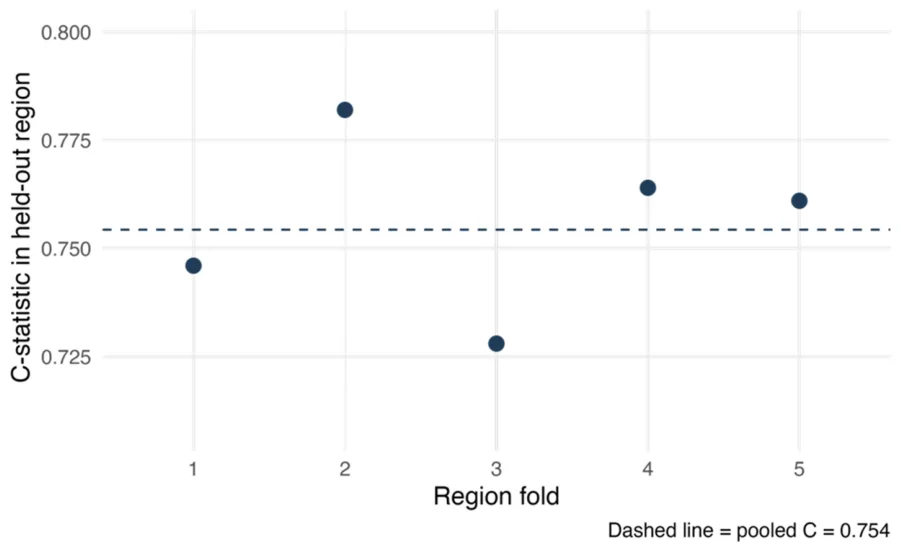
Internal–External Validation by Held-Out Geographic Region. *Note.* The 27 provinces represented in the analytic sample were grouped into five geographic folds. Each point is the C-statistic obtained in a fold omitted from model development, so no regional estimate came from a model trained on participants from that region. The dashed line indicates the pooled C-statistic across held-out predictions.

The pooled internal–external C-statistic was 0.754. C-statistics in the five held-out geographic folds were 0.746, 0.782, 0.728, 0.764, and 0.761 (Table 5 and Figure 7). The pooled estimate was therefore similar to the principal cross-validated estimate, and all held-out estimates remained within the moderate-discrimination range. Discrimination was therefore not dependent on the inclusion of any single geographic fold during model development. The observed range indicates some regional variation, and no formal heterogeneity estimate or region-specific calibration was calculated.

## 4. Discussion

In a large, nationally representative community cohort of middle-aged and older adults who were symptomatic at the landmark, sustained remission of depressive symptoms was moderately predictable from routinely collected information, and most of the prognostic information was concentrated in a small, interpretable set of factors. This study makes several contributions. First, recent symptom trajectory was the dominant contributor, extending to remission a pattern previously observed in research on incident depression and on relapse or recurrence. Second, pain was the only factor outside the symptom trajectory to improve prediction, although its incremental contribution was modest. Third, behavioral, psychosocial, and sociodemographic variables added no appreciable independent prognostic information once the symptom trajectory and pain were known. Fourth, the model was well calibrated, and its discrimination was maintained when whole regions were held out.

### 4.1. Prior Depressive Symptoms and Sustained Remission

The dominance of symptom history is consistent with evidence that prior depressive history, residual symptoms, and symptom severity are associated with the subsequent course of depression [15,16]. Related prospective work has also identified subsyndromal symptoms, previous depression, and functional impairment as predictors of incident major depressive episodes among older primary-care patients [8]. The present findings indicate that recent symptom trajectory also predicts whether elevated symptoms remit in a community population in which formal treatment is uncommon. This extends the prognostic relevance of symptom history to naturalistic remission, although it does not establish that identical mechanisms operate across incident, remitted, treated, and community samples. Several mechanisms may plausibly contribute. Residual and subthreshold symptoms may indicate that the underlying depressive process remains active rather than representing a benign state, and they predict a more rapid return of major depressive symptoms among people who have otherwise improved [16]. Kindling and stress-sensitization accounts further propose that, across repeated or sustained episodes, the relationship between external stressors and depressive episodes may change, with progressively less stress required to precipitate subsequent episodes [48]. In a population context in which treatment coverage is low [5], symptom history may therefore capture aspects of an illness course that have not been substantially modified by formal intervention. Because individual treatment use was not established comprehensively, the cohort should be characterized as largely rather than uniformly untreated.

### 4.2. Regression to the Mean and the Availability of Symptom History

Two features of the design require cautious interpretation. Because eligibility and the outcome were defined using the same instrument, part of the predictive value of symptom severity may reflect regression to the mean, whereby participants scoring near the eligibility threshold at the landmark are more likely to fall below it on remeasurement. To examine this possibility, we restricted the sample to participants with more severe or persistent symptoms, for whom threshold-related regression should be reduced. Discrimination attenuated but remained moderate, and the model continued to distinguish participants who remitted from those who did not. Regression to the mean may therefore account for some, but not all, of the observed discrimination. Pain, which was measured independently of the depressive-symptom instrument, also contributed prognostic information. The design nevertheless cannot fully separate the prognostic contribution of illness course from residual measurement dependence. Future research could establish eligibility through a diagnostic interview and assess remission using a separate symptom scale, and more frequent assessments would allow the intervening course to be observed rather than inferred from widely separated measurements.

Although the strongest model drew on several years of prior symptom history, such information may not always be available in community or primary-care practice. Routine implementation may be constrained by brief consultations and weak informational continuity in Chinese primary care. In Beijing community-health institutions, the median general-practice consultation lasted 2 min and history taking occurred in only 27% of visits, and a national review found primary-care electronic records to be commonly unavailable, fragmented, or isolated [49,50]. Predictors requiring several years of symptom history may therefore be difficult to ascertain reliably during routine care. We therefore fitted a restricted model using information obtainable at a single assessment, comprising the current depressive-symptom score, pain, and demographic characteristics. This model discriminated less well than the full model, although it retained moderate discrimination and remained well calibrated. It is therefore more representative of what might be achieved from a single clinical encounter. The full model is better understood as identifying where the available prognostic information resides than as a tool ready for immediate implementation.

### 4.3. Pain and Sustained Remission

Pain was the principal predictor of reduced remission outside the symptom trajectory. Penalization retained both the presence of pain and the number of affected body sites while shrinking most other physical-health variables. Adding the physical-health block to the illness-course block produced a small improvement in discrimination, and a parsimonious model containing illness-course and pain measures performed similarly to the full 54-predictor model. Pain therefore appears to provide genuine but modest prognostic information beyond recent depressive symptoms. This finding is consistent with the well-documented comorbidity of pain and depression [17] and with evidence of reciprocal longitudinal relationships in aging cohorts, in which pain may predict later depressive symptoms and depressive symptoms may predict later pain [18]. A behavioral pathway offers one plausible explanation. Under the activity-restriction model, pain may interfere with valued and routine activities, and the resulting reduction in engagement and positive reinforcement may contribute to depressive symptoms [51]. The independent signal associated with the number of pain sites is consistent with this interpretation, because more widespread pain may impose broader activity restrictions, although the present data cannot establish it. Pain should therefore be interpreted as a prognostic marker rather than an established modifiable cause. The available measures captured the presence and number of pain sites but not intensity, duration, pain-related interference, or the distinction between acute and chronic pain, all of which are important dimensions of pain assessment [52]. The observational design also cannot establish that reducing pain would increase the probability of remission. Whether pain management improves depressive outcomes in this population requires direct evaluation in intervention studies. Within these limits, the ease with which the two pain indicators can be collected supports their potential value as practical prognostic signals.

### 4.4. Behavioral, Psychosocial, and Sociodemographic Factors and Sustained Remission

The absence of additional predictive improvement from the behavioral, psychosocial, and sociodemographic blocks also requires careful interpretation. Socioeconomic disadvantage, physical inactivity, and poor social relationships are established correlates of prevalent or incident depression [19,20,21]. The present outcome, however, was remission conditional on already being symptomatic, and factors associated with developing a condition need not have the same prognostic importance after that condition has been established [12]. Their lack of incremental prediction does not imply that these factors are unimportant determinants of mental health or inappropriate targets for population-level intervention. Several variables, including smoking, alcohol consumption, internet use, and household wealth, also had small positive coefficients in directions that would be difficult to interpret as causal protective effects. These patterns may reflect residual confounding, because health behaviors are socially patterned by age, sex, and socioeconomic position, and socioeconomic disadvantage is itself associated with depression [19]. The defensible interpretation is therefore predictive rather than etiological. Beyond recent symptom trajectory and pain, the available community-survey variables provided little additional independent information for predicting remission.

### 4.5. Model Discrimination and Clinical Interpretation

The moderate discrimination achieved should be interpreted in the context of the broader prediction literature. Reviews have found modest performance for machine-learning models of depression treatment outcomes, few externally validated models of relapse or sustained recovery, and substantial risks of bias across psychiatric prediction-model studies [9,10,53]. The practical value of a moderately discriminating model consequently lies more plausibly in risk-group stratification than in confident prediction for an individual person [54]. In a low-treatment context, such stratification could help identify groups less likely to remit without support and inform closer monitoring or service prioritization, consistent with stepped and stratified models of care [23,24]. Any use for resource allocation would nevertheless require evaluation of clinical utility, fairness, feasibility, and the consequences of false-positive and false-negative classifications.

### 4.6. Geographic Transportability

The internal–external validation examined whether discrimination was maintained when entire geographic folds were omitted from model development. Held-out C-statistics ranged from 0.728 to 0.782, with a pooled value of 0.754, and all remained within the moderate-discrimination range. These findings indicate that the model’s discrimination was not dependent on the inclusion of any single geographic fold during development. This geographic consistency is relevant because national studies have demonstrated substantial inequity in mental-health-bed allocation and persistent urban–rural and east–west differences in the mental-health workforce [13,14]. Internal–external validation provides stronger evidence of geographic transportability than ordinary random-split internal validation [29,30]. Nevertheless, validation and, where necessary, recalibration in an independent cohort remain necessary before clinical implementation. Furthermore, because imputation was completed before resampling and was not repeated when geographic folds were held out, the internal–external results are conditional on the completed datasets similar to the primary validation estimates. The apparent stability of discrimination across folds should therefore be read as evidence that performance did not depend on any single group of provinces, rather than as evidence that the full preprocessing and model-development pipeline transports unchanged.

### 4.7. Strengths and Limitations

This study used a large, nationally representative cohort, prespecified a comprehensive candidate set, employed penalization with clustered resampling, and evaluated calibration and geographic transportability in addition to discrimination. Several limitations qualify the findings.

Discrimination was moderate, and part of the signal may reflect regression to the mean, so the model is better suited to cautious risk stratification than to deterministic individual prediction. Anxiety, which is among the more consistent predictors of an adverse depressive course, was not assessed in CHARLS and may contribute to the model’s performance ceiling [15,55]. Depressive symptoms were measured by self-report using the CES-D-10 rather than by clinical interview, and the somatic content of symptom scales may overlap conceptually with physical-health predictors, requiring caution when interpreting those associations. The cutoff of 10 is a screening threshold rather than a diagnosis, and validation research among older Chinese adults has proposed a higher threshold when diagnostic classification is the objective [32,56]. Alternative thresholds and clinically established diagnoses should therefore be examined in future validation studies. The pain measures were coarse, and the outcome represented remission at two assessment waves rather than continuously observed remission.

Attrition may have influenced the findings. Excluded participants were older and more functionally impaired than those retained, indicating that outcome availability was patterned by variables plausibly associated with remission [57]. We therefore examined robustness under two conservative sensitivity scenarios. First, redefining the favorable outcome as being alive and in sustained remission reduced the remission proportion from 19.9% to 18.7%, while discrimination remained essentially unchanged (C-statistic = 0.759 versus 0.755). This composite endpoint appropriately treated recorded death as precluding sustained remission rather than imputing a symptom outcome after death [34,35]. Second, classifying all 2239 excluded participants as non-remitters produced a C-statistic of 0.750 for the full model and 0.704 for the single-contact model. The consistency of these estimates indicates that the model’s discrimination was not materially driven by recorded mortality or by favorable outcomes assigned to participants lost to follow-up. Inverse-probability-of-attrition weighting was not additionally applied because it would introduce a separate model-dependent assumption that the probability of outcome observation was correctly specified [58]. The sensitivity analyses therefore support the robustness of discrimination, although attrition may still affect absolute-risk calibration and representativeness. Because deaths were identified only through the 2018 wave, the mortality analysis did not capture all deaths that may have occurred before the 2020 assessment.

Several analytic constraints followed from using an existing cohort. Because all eligible CHARLS participants were included, sample size was fixed by the available data rather than determined prospectively, and its adequacy was contingent on the anticipated model performance rather than established in advance. Imputation was performed once, with resampling subsequently conducted within the imputed datasets rather than imputation being repeated during every resampling iteration. Because most of the missing prior-wave data are structural, arising because refreshment-sample members were never surveyed in the earlier waves, repeated imputation would characterize variability in values that could in principle have been observed rather than addressing that structural gap. A complete-case comparison tests the concern that applies here more directly, and discrimination was not lower in that subset. Imputation uncertainty is nonetheless not fully propagated into the optimism and interval estimates. We partly addressed this by deriving confidence intervals from a household bootstrap of pooled out-of-fold predictions. Cohorts with harmonized recruitment across waves, or linkage to routine records predating cohort entry, would allow prior symptom history to be observed rather than imputed.

The reciprocal relationship between pain and depression precludes causal interpretation. The analysis used one cohort with internal and internal–external validation, and validation in an independent cohort is required before implementation [26,59]. Treatment coverage for depressive disorders is low in China, suggesting that the findings may predominantly reflect a naturalistic or untreated course, although individual treatment exposure was not comprehensively established. The findings may therefore be less transportable to settings in which treatment is more widely available. Finally, the data predate 2021, and applicability to more recent populations and service contexts should be confirmed.

### 4.8. Directions for Future Research

Future research should investigate whether information omitted from many community surveys, including anxiety, detailed treatment exposure, finer-grained symptom dynamics, pain intensity and interference, and potentially relevant biological markers, improves predictive performance. The model should also be externally validated and recalibrated where necessary. Intervention research is required to determine whether identifying people at lower probability of remission improves outcomes and whether effective pain management contributes to the remission of depressive symptoms.

## 5. Conclusions

Sustained remission of depressive symptoms was moderately and consistently predictable from routinely collected data in this community cohort. Prognostic information was driven principally by recent symptom trajectory and, secondarily, by pain, with little incremental contribution from the available behavioral, social, or economic variables. A model restricted to single-visit information discriminated less well, and some of the apparent signal may reflect regression to the mean. The model should therefore be positioned as a candidate tool for cautious prognostic stratification rather than for individual-level prediction. Recent symptom history and the presence and distribution of bodily pain may provide a low-cost basis for identifying symptomatic middle-aged and older adults who warrant closer monitoring or additional assessment. Consistent discrimination across held-out geographic folds supports potential geographic transportability within China, but independent external validation, evaluation of clinical utility and fairness, and research examining whether pain management improves remission remain necessary before implementation.

## Figures and Tables

**Table 1:** Characteristics of the Analytic Sample by Sustained Remission.

Predictor	Non-Remitters (n = 3299)	Remitters (n = 818)	*p*	Missing (%)
*Illness course*				
CES-D score, 2015 (landmark)	15.75 (4.74)	13.17 (3.48)	<0.001	0.0
CES-D score, 2013	12.24 (6.06)	8.19 (5.03)	<0.001	21.9
CES-D score, 2011	12.44 (6.55)	8.30 (5.77)	<0.001	29.6
Elevated symptoms, 2013, n (%)	1662 (63.8)	203 (33.4)	<0.001	21.9
Elevated symptoms, 2011, n (%)	1519 (64.2)	198 (37.0)	<0.001	29.6
Symptom change, 2013–2015	3.61 (6.26)	4.97 (5.50)	<0.001	21.9
Prior waves with elevated symptoms	1.13 (0.76)	0.61 (0.69)	<0.001	15.7
Somatic subscale	10.40 (2.61)	9.24 (2.25)	<0.001	0.0
Affective subscale	9.58 (2.76)	8.55 (2.21)	<0.001	0.0
*Physical health*				
Chronic conditions	1.88 (1.59)	1.35 (1.35)	<0.001	0.0
ADL limitations	0.63 (1.15)	0.32 (0.84)	<0.001	1.2
IADL limitations	0.68 (1.13)	0.32 (0.76)	<0.001	0.0
Mobility limitation	2.29 (1.87)	1.48 (1.62)	<0.001	0.0
Body mass index	23.85 (3.90)	23.68 (3.50)	0.298	15.1
Cognition composite	13.63 (4.55)	14.91 (4.40)	<0.001	17.8
Pain present, n (%)	1947 (59.0)	308 (37.7)	<0.001	0.0
Pain site count	3.43 (3.96)	1.74 (2.95)	<0.001	0.0
Distance vision	4.04 (0.90)	3.80 (0.96)	<0.001	0.3
Near vision	3.97 (0.91)	3.80 (0.92)	<0.001	0.3
Hearing problem, n (%)	275 (9.4)	65 (8.6)	0.512	10.7
Any fall, n (%)	835 (25.3)	137 (16.7)	<0.001	0.0
Childhood health	2.86 (1.17)	2.75 (1.12)	0.013	1.3
Self-rated health, 2013	3.35 (0.87)	2.98 (0.87)	<0.001	20.0
*Lifestyle*				
Ever smoked, n (%)	1128 (34.2)	359 (43.9)	<0.001	0.0
Current smoker, n (%)	764 (23.2)	241 (29.5)	<0.001	0.0
Any drinking, n (%)	954 (28.9)	305 (37.3)	<0.001	0.1
Drinking frequency	1.20 (2.39)	1.64 (2.67)	<0.001	0.4
Vigorous activity, n (%)	686 (41.7)	150 (37.2)	0.117	50.2
Moderate activity, n (%)	998 (60.6)	240 (59.7)	0.786	50.2
Light activity, n (%)	1323 (80.2)	324 (80.6)	0.924	50.2
Sleep duration, hours	5.72 (2.08)	6.14 (1.81)	<0.001	1.1
*Psychosocial and family*				
Social activity count	0.85 (1.06)	1.00 (1.21)	<0.001	0.0
Interacting with friends, n (%)	1134 (34.4)	302 (36.9)	0.185	0.0
Games or club, n (%)	526 (15.9)	154 (18.8)	0.053	0.0
Helping others, n (%)	532 (16.1)	135 (16.5)	0.834	0.0
Sport or social club, n (%)	227 (6.9)	63 (7.7)	0.456	0.0
Volunteering, n (%)	38 (1.2)	14 (1.7)	0.268	0.0
Educational activity, n (%)	12 (0.4)	7 (0.9)	0.116	0.0
Internet use, n (%)	101 (3.1)	65 (7.9)	<0.001	0.0
Any activity almost daily, n (%)	720 (21.8)	202 (24.7)	0.086	0.0
Partnered, n (%)	2842 (86.1)	724 (88.5)	0.086	0.0
Became widowed, 2013–2015, n (%)	92 (3.4)	7 (1.1)	0.003	18.2
Child co-resident, n (%)	1891 (58.1)	487 (60.2)	0.291	1.3
Child lives nearby, n (%)	2739 (85.6)	684 (85.9)	0.853	2.9
Number of living children	2.70 (1.30)	2.45 (1.21)	<0.001	0.0
Living siblings	3.62 (1.83)	3.58 (1.79)	0.633	2.5
Money received from children (log)	4.82 (3.98)	4.39 (4.13)	0.033	39.2
Net transfer (log)	2.01 (6.98)	0.94 (7.32)	0.002	39.1
*Sociodemographic*				
Age, years	58.18 (9.16)	56.88 (9.51)	<0.001	0.1
Female, n (%)	2177 (66.0)	434 (53.1)	<0.001	0.0
Education level	1.07 (0.27)	1.10 (0.35)	0.001	0.0
Rural hukou, n (%)	2567 (87.5)	584 (81.9)	<0.001	11.4
Household wealth (log)	7.57 (8.03)	8.90 (7.05)	<0.001	35.0
Any wage income, n (%)	525 (15.9)	179 (22.0)	<0.001	0.2

*Note.* Sustained remission was defined as a CES-D-10 score below 10 at both the 2018 and 2020 assessments. Continuous variables are presented as mean (SD) and binary variables as n (%). Percentages are calculated among participants with non-missing data, so denominators differ where missingness is present. The *p*-values are from unadjusted comparisons and are descriptive; they were not used for predictor selection. Vision, childhood health, and self-rated health are coded so that higher values indicate poorer status. CES-D = Center for Epidemiologic Studies Depression Scale; ADL = activities of daily living; IADL = instrumental activities of daily living.

**Table 2:** Univariable and Penalized Multivariable Associations With Sustained Remission.

Predictor	Univariable OR [95% CI]	*p*	Penalized Multivariable OR
*Illness course*			
CES-D score, 2015	0.48 [0.43, 0.54]	<0.001	0.71
CES-D score, 2013	0.44 [0.39, 0.50]	<0.001	0.72
CES-D score, 2011	0.48 [0.43, 0.54]	<0.001	0.74
Elevated symptoms, 2013	0.29 [0.24, 0.34]	<0.001	0.95
Elevated symptoms, 2011	0.33 [0.27, 0.40]	<0.001	1.00
Symptom change, 2013–2015	1.25 [1.15, 1.36]	<0.001	Not selected
Prior waves with elevated symptoms	0.48 [0.44, 0.53]	<0.001	0.92
Somatic subscale	0.62 [0.57, 0.67]	<0.001	Not selected
Affective subscale	0.66 [0.62, 0.72]	<0.001	Not selected
*Physical health*			
Chronic conditions	0.69 [0.63, 0.75]	<0.001	0.96
ADL limitations	0.68 [0.61, 0.77]	<0.001	Not selected
IADL limitations	0.63 [0.56, 0.71]	<0.001	0.92
Mobility limitation	0.61 [0.56, 0.67]	<0.001	0.94
Body mass index	0.96 [0.88, 1.04]	0.266	0.97
Cognition composite	1.34 [1.23, 1.46]	<0.001	1.08
Pain present	0.42 [0.36, 0.49]	<0.001	0.92
Pain site count	0.58 [0.53, 0.64]	<0.001	0.92
Distance vision	0.78 [0.73, 0.84]	<0.001	0.97
Near vision	0.83 [0.78, 0.90]	<0.001	1.00
Hearing problem	0.90 [0.68, 1.20]	0.469	1.02
Any fall	0.59 [0.49, 0.72]	<0.001	0.98
Childhood health	0.91 [0.84, 0.98]	0.012	0.98
Self-rated health, 2013	0.66 [0.60, 0.72]	<0.001	0.92
*Lifestyle*			
Ever smoked	1.50 [1.29, 1.76]	<0.001	1.06
Current smoker	1.38 [1.17, 1.64]	<0.001	Not selected
Any drinking	1.46 [1.24, 1.72]	<0.001	1.01
Drinking frequency	1.18 [1.10, 1.26]	<0.001	Not selected
Vigorous activity	0.83 [0.66, 1.04]	0.105	0.93
Moderate activity	0.96 [0.77, 1.21]	0.747	0.98
Light activity	1.02 [0.78, 1.35]	0.869	1.00
Sleep duration	1.23 [1.14, 1.32]	<0.001	Not selected
*Psychosocial and family*			
Social activity count	1.14 [1.06, 1.23]	<0.001	Not selected
Interacting with friends	1.12 [0.95, 1.31]	0.172	Not selected
Games or club	1.22 [1.00, 1.49]	0.046	0.98
Helping others	1.03 [0.84, 1.26]	0.791	0.99
Sport or social club	1.13 [0.84, 1.51]	0.412	1.00
Volunteering	1.49 [0.80, 2.77]	0.203	1.00
Educational activity	2.36 [0.93, 6.02]	0.071	1.00
Internet use	2.73 [1.99, 3.76]	<0.001	1.06
Any activity almost daily	1.17 [0.98, 1.40]	0.076	Not selected
Partnered	1.24 [0.98, 1.57]	0.077	0.96
Became widowed, 2013–2015	0.32 [0.15, 0.69]	0.004	0.88
Child co-resident	1.09 [0.93, 1.28]	0.278	Not selected
Child lives nearby	1.03 [0.82, 1.29]	0.810	1.01
Number of living children	0.81 [0.74, 0.88]	<0.001	0.93
Living siblings	0.98 [0.91, 1.06]	0.629	Not selected
Money received from children (log)	0.90 [0.82, 0.99]	0.036	1.00
Net transfer (log)	0.86 [0.78, 0.95]	0.003	0.97
*Sociodemographic*			
Age	0.87 [0.80, 0.94]	<0.001	1.00
Female	0.58 [0.50, 0.68]	<0.001	0.93
Education level	1.12 [1.04, 1.20]	0.001	1.00
Rural hukou	0.65 [0.52, 0.81]	<0.001	0.97
Household wealth (log)	1.21 [1.08, 1.36]	<0.001	1.05
Any wage income	1.48 [1.23, 1.80]	<0.001	1.00

*Note.* Univariable odds ratios were estimated using logistic regression with household-clustered standard errors and are expressed per standard deviation for continuous predictors; they may differ slightly from the unadjusted comparisons in Table 1, which use chi-square tests for binary variables. The *p*-values are descriptive and were not used to select candidate predictors. Multivariable odds ratios are exponentiated elastic-net coefficients averaged across 30 completed datasets. Conventional confidence intervals were not calculated for penalized estimates. “Not selected” denotes a coefficient reduced exactly to zero at the selected penalty. Selection is conditional on the other predictors and the penalty. OR = odds ratio; CI = confidence interval.

**Table 3:** Model Performance and Internal Validation.

Measure	Full Model	Parsimonious Model	Gradient Boosting
Apparent C-statistic	0.774	—	—
Estimated optimism	0.012	—	—
Optimism-corrected C-statistic	0.761	—	—
Cross-validated C-statistic	0.755	0.755	0.735
Calibration slope	1.06	1.02	0.78
Calibration-in-the-large	0.00	0.00	—
Brier score	0.138	0.137	0.142
Pre-pandemic 2018 C-statistic	0.715	—	—

*Note.* Apparent and optimism-corrected discrimination were estimated using 500 household-cluster bootstrap resamples. Cross-validated performance was based on pooled out-of-fold predictions across 30 completed datasets. The 95% confidence interval for the full-model cross-validated C-statistic was [0.749, 0.784]. Imputation was completed before and not repeated within resampling; validation estimates are therefore conditional on the completed datasets. The parsimonious model comprised the illness course and pain predictors. Dashes denote measures that were not estimated for that model rather than measures that are inapplicable: apparent performance, optimism, optimism-corrected discrimination, and the pre-pandemic analysis were computed only for the full model, and calibration-in-the-large was not computed for the gradient-boosting comparator.

**Table 4:** Sensitivity Analyses of Discrimination.

Analysis	n	Remission Events	C-Statistic
Primary full model	4117	818	0.755
CES-D ≥ 12 at landmark	3089	486	0.754
CES-D ≥ 15 at landmark	1919	215	0.721
Elevated symptoms in both 2013 and 2015	1865	203	0.706
Single-contact model	4117	818	0.698
Complete prior-wave data	2645	485	0.764
Alive and in sustained remission	4382	818	0.759
All excluded participants as non-remitters	6356	818	0.750

*Note.* Values are pooled cross-validated C-statistics averaged across completed datasets. The single-contact model comprised the 2015 CES-D-10 score, pain presence, pain-site count, age, sex, education, and household registration. In the “alive and in sustained remission” analysis, participants known to have died were classified as not achieving the favorable composite outcome. The final analysis represents an extreme outcome-classification sensitivity analysis rather than an empirically supported missing-outcome mechanism.

**Table 5:** Internal–External Validation by Geographic Region.

Held-Out Geographic Fold	C-Statistic
1	0.746
2	0.782
3	0.728
4	0.764
5	0.761
Pooled	0.754

*Note.* The 27 provinces represented in the sample were grouped into five geographic folds. Each C-statistic was obtained in a fold omitted from model development. The pooled value summarizes discrimination across held-out predictions. The range is descriptive because a formal estimate of between-region heterogeneity was not calculated.

## Data Availability

The data analyzed in this study are publicly available from the China Health and Retirement Longitudinal Study and can be accessed at https://charls.pku.edu.cn/ (accessed on 26 June 2026) following registration. The analysis code is available from the corresponding author upon reasonable request.

## Supplementary Materials

The following supporting information can be downloaded at: https://www.mdpi.com/article/10.3390/healthcare14183112/s1, Table S1: Definitions and Coding of Candidate Predictors; Table S2: Rationale for Predictor Blocks; Table S3: Full Model Specification; Table S4: Comparison of Included and Excluded Participants; Table S5: Bootstrap Selection Frequencies; Table S6: Composition of the Folds Used in Internal-External Validation; Figure S1: Iteration Trace Plots for Multiple Imputation by Chained Equations; TRIPOD + AI Checklist. Ref. [28] is cited in Supplementary Materials.
