# Supplementary material for: Development and Internal Validation of a Multivariable Prognostic Model for Sustained Remission of Depressive Symptoms in Middle-Aged and Older Adults: An Analysis of the CHARLS Cohort
Source: Healthcare (Basel). 2026 Sep 21;14(18):3112. doi: 10.3390/healthcare14183112 (PMC13606152; doi:10.3390/healthcare14183112)
Supplement: Supplementary file 1 [file healthcare-14-03112-s001.zip › healthcare-4539867-supplementary.pdf]

Supplementary Materials

Table S1

Definitions and Coding of Candidate Predictors

Variable	Coding and category labels
Illness course	
Index CES-D-10, 2015	Total score, 0-30 (continuous)
Prior CES-D-10, 2013	Total score, 0-30 (continuous)
Cohort-baseline CES-D-10, 2011	Total score, 0-30 (continuous)
Elevated symptoms, 2013	1 = CES-D-10 ≥ 10 in 2013; 0 = otherwise
Elevated symptoms, 2011	1 = CES-D-10 ≥ 10 in 2011; 0 = otherwise
Symptom change, 2013-2015	2015 score minus 2013 score (continuous)
Prior waves with elevated symptoms	Count of waves, 0-2
Somatic subscale	Sum of CES-D-10 somatic items (continuous)
Affective subscale	Sum of CES-D-10 affective items (continuous)
Physical health	
Chronic conditions	Count of physician-diagnosed conditions, 0-9
ADL limitations	Count, 0-5
IADL limitations	Count, 0-5
Mobility limitation	Count of gross-motor limitations
Body mass index	kg/m^2 (continuous)
Cognition composite	Global cognition score (continuous)
Pain present	1 = often troubled by bodily pain; 0 = not
Pain site count	Number of body sites with reported pain, 0-13
Distance vision	1 = excellent to 5 = poor
Near vision	1 = excellent to 5 = poor
Hearing problem	1 = yes; 0 = no
Any fall	1 = fell in the past two years; 0 = no
Childhood health	1 = excellent to 5 = poor
Self-rated health, 2013 (lagged)	1 = very good to 5 = very poor
Lifestyle	
Ever smoked	1 = yes; 0 = no
Current smoker	1 = yes; 0 = no
Any drinking	1 = yes; 0 = no

Variable	Coding and category labels
Drinking frequency	Ordinal frequency category, 0-9
Vigorous activity	1 = yes; 0 = no
Moderate activity	1 = yes; 0 = no
Light activity	1 = yes; 0 = no
Sleep duration	Hours per night (continuous)
Psychosocial and family	
Social activity count	Count of social activities endorsed, 0-7
Interacting with friends	1 = yes; 0 = no
Games or club	1 = yes; 0 = no
Helping others	1 = yes; 0 = no
Sport or social club	1 = yes; 0 = no
Volunteering	1 = yes; 0 = no
Educational activity	1 = yes; 0 = no
Internet use	1 = yes; 0 = no
Any activity almost daily	1 = yes; 0 = no
Partnered (recoded)	1 = married or cohabiting; 0 = separated, divorced, widowed, or never married
Became widowed, 2013-2015	1 = yes; 0 = no
Child co-resident	1 = yes; 0 = no
Child lives nearby	1 = yes; 0 = no
Number of living children	Count
Living siblings	Count
Money received from children	CNY, signed-logarithmic transformation (continuous)
Net transfer	Net CNY to or from children, signed-logarithmic transformation (continuous)
Sociodemographic	
Age	Years (continuous)
Female	1 = female; 0 = male
Education	1 = less than lower secondary; 2 = upper secondary or vocational; 3 = tertiary
Rural hukou (recoded)	1 = rural (agricultural); 0 = urban (non-agricultural)
Household wealth	CNY, signed-logarithmic transformation (continuous)
Any wage income	1 = yes; 0 = no

Note. All predictors were measured at or before the 2015 landmark. Self-rated health was entered using its 2013 value to ensure it preceded the landmark. Count and ordinal variables were entered

as single linear terms, and all predictors were standardized to a mean of zero and standard deviation of one before penalized estimation. For the two nominal variables, marital status and household registration, the recoded binary form shown here was used. The education variable was highly skewed in the analytic sample, with 93.3% of participants in the lowest category. CES-D-10 = 10-item Center for Epidemiologic Studies Depression Scale; ADL = activities of daily living; IADL = instrumental activities of daily living.

Table S2

Rationale for Predictor Blocks

Block	Predictors	Rationale
Illness course	CES-D severity in 2015, 2013, and 2011; prior-wave elevated-symptom status; symptom change; prior elevated-symptom waves; somatic and affective subscales	Symptom severity, residual symptoms, and previous depressive history are among the most consistent prognostic factors for depressive course (Buckman et al., 2018; Judd et al., 1998).
Physical health	Comorbidity; ADL and IADL limitations; mobility; body mass index; cognition; pain presence and site count; vision; hearing; falls; childhood health; lagged self-rated health	Physical illness, disability, and pain are associated with depression, and pain shows reciprocal longitudinal associations with depressive symptoms in aging cohorts (Bair et al., 2003; Qiu et al., 2022).
Lifestyle	Smoking; alcohol consumption; vigorous, moderate, and light physical activity; sleep duration	Physical activity and other health behaviors have been associated with incident depression and were examined as potentially informative routinely collected predictors (Schuch et al., 2018).
Psychosocial and family	Social activities; marital status; widowhood; living arrangements; living children and siblings; intergenerational transfers	Social relationships and support are associated with depression in later life (Schwarzbach et al., 2014).
Sociodemographic	Age; sex; education; household registration; household wealth; wage income	Socioeconomic and demographic characteristics are associated with depression in community populations and are routinely available for prognostic assessment (Lorant et al., 2003).

Note. Blocks were specified before model fitting. All 54 predictors were entered simultaneously. The block structure was used to characterize the incremental contribution of each domain in the sequential analysis. ADL = activities of daily living; IADL = instrumental activities of daily living; CES-D = Center for Epidemiologic Studies Depression Scale.

Table S3

Full Model Specification

Predictor	Standardized coefficient	Mean	SD
(Intercept)	−1.7043	—	—
Illness course			
Index CES-D, 2015	−0.3377	15.2366	4.6331
Prior CES-D, 2013	−0.3265	11.2933	6.0472
Cohort-baseline CES-D, 2011	−0.2952	11.3182	6.5847
Elevated symptoms, 2013	−0.0479	0.5680	0.4954
Elevated symptoms, 2011	−0.0001	0.5686	0.4953
Symptom change, 2013-2015	0 (not selected)	3.9433	6.0965
Prior waves with elevated symptoms	−0.0862	1.1366	0.7941
Somatic subscale	0 (not selected)	10.1725	2.5861
Affective subscale	0 (not selected)	9.3719	2.6931
Physical health			
Chronic conditions	−0.0423	1.7712	1.5559
ADL limitations	0 (not selected)	0.5632	1.0970
IADL limitations	−0.0815	0.6077	1.0728
Mobility limitation	−0.0613	2.1285	1.8486
Body mass index	−0.0322	23.8234	3.8251
Cognition composite	0.0778	13.6420	4.5886
Pain present	−0.0811	0.5477	0.4978
Pain site count	−0.0855	3.0918	3.8420
Distance vision	−0.0268	3.9960	0.9191
Near vision	−0.0001	3.9396	0.9134
Hearing problem	0.0202	0.0969	0.2958
Any fall	−0.0152	0.2361	0.4247
Childhood health	−0.0229	2.8403	1.1575
Self-rated health, 2013	−0.0786	3.2219	0.8903
Lifestyle			
Ever smoked	0.0582	0.3612	0.4804
Current smoker	0 (not selected)	0.2443	0.4297
Any drinking	0.0107	0.3060	0.4609
Drinking frequency	0 (not selected)	1.2942	2.4565

Predictor	Standardized coefficient	Mean	SD
Vigorous activity	−0.0679	0.4023	0.4903
Moderate activity	−0.0226	0.5992	0.4901
Light activity	0.0039	0.7989	0.4008
Sleep duration	0 (not selected)	5.8013	2.0404
Psychosocial and family			
Social activity count	0 (not selected)	0.8790	1.0955
Interacting with friends	0 (not selected)	0.3488	0.4766
Games or club	−0.0171	0.1652	0.3714
Helping others	−0.0073	0.1620	0.3685
Sport or social club	−0.0021	0.0704	0.2559
Volunteering	0.0010	0.0126	0.1117
Educational activity	0.0002	0.0046	0.0678
Internet use	0.0582	0.0403	0.1967
Any activity almost daily	0 (not selected)	0.2239	0.4169
Partnered	−0.0359	0.8662	0.3405
Became widowed, 2013-2015	−0.1247	0.0286	0.1666
Child co-resident	0 (not selected)	0.5837	0.4930
Child lives nearby	0.0055	0.8498	0.3573
Number of living children	−0.0722	2.6541	1.2900
Living siblings	0 (not selected)	3.6062	1.8259
Money received from children (log)	−0.0037	4.6100	4.0286
Net transfer (log)	−0.0316	1.4685	7.0751
Sociodemographic			
Age	0.0008	57.9230	9.2449
Female	−0.0704	0.6342	0.4817
Education level	−0.0038	1.0746	0.2883
Rural hukou	−0.0300	0.8616	0.3453
Household wealth (log)	0.0505	7.8623	7.8409
Any wage income	0.0003	0.1715	0.3769

Note. Coefficients are penalized elastic-net estimates on the standardized scale, averaged across 30 imputed datasets, and express the change in the log-odds of sustained remission per standard deviation of the predictor. To apply the averaged model to a new individual, subtract the reported mean from each raw predictor value, divide by the reported standard deviation, multiply by the corresponding coefficient, sum across predictors, add the intercept, and transform the linear predictor using the inverse-logit function. Means and standard deviations are those of the imputed

development sample and were averaged across imputations. Because coefficients and standardization constants were averaged across imputation-specific models, this single specification may not reproduce exactly the average of predictions from all 30 original models. Predictors with coefficients of exactly zero contribute nothing to the prediction. Binary predictors are coded 0 or 1, and their means represent the corresponding proportions in the development sample. Household wealth and intergenerational transfers were entered after signed-logarithmic transformation, and self-rated health was lagged to 2013; values must undergo the same transformations before standardization. The model requires prior-wave CES-D scores and therefore cannot be applied directly when these historical values are unavailable. CES-D = Center for Epidemiologic Studies Depression Scale; SD = standard deviation; ADL = activities of daily living; IADL = instrumental activities of daily living.

Table S4

Comparison of Included and Excluded Participants

Characteristic	Included (n = 4,117)	Excluded (n = 2,239)
Age, years	57.92 (9.24)	62.86 (11.16)
CES-D score, 2015	15.24 (4.63)	15.73 (4.85)
Chronic conditions	1.77 (1.56)	1.99 (1.67)
ADL limitations	0.57 (1.10)	0.82 (1.32)
IADL limitations	0.61 (1.07)	0.97 (1.37)
Mobility limitation	2.13 (1.85)	2.64 (2.07)
Pain present, %	54.8	56.2
Pain site count	3.09 (3.84)	3.12 (3.84)
Female, %	63.4	62.1

Note. Values are mean (SD) unless otherwise stated. Excluded participants lacked one or both follow-up assessments; 265 were known to have died by the 2018 wave. All characteristics were measured at the 2015 landmark. CES-D = Center for Epidemiologic Studies Depression Scale; ADL = activities of daily living; IADL = instrumental activities of daily living.

Table S5

Bootstrap Selection Frequencies

Predictor	Retained (%)
CES-D score, 2015	100.0
CES-D score, 2013	100.0
CES-D score, 2011	100.0
Became widowed, 2013-2015	99.8
Vigorous activity	98.8
Cognition composite	97.6
Self-rated health, 2013	97.6
IADL limitations	95.6
Number of living children	93.8
Pain present	92.6
Internet use	91.6
Prior waves with elevated symptoms	90.0
Pain site count	89.8
Body mass index	88.0
Female	87.0
Moderate activity	85.2
Ever smoked	82.2
Mobility limitation	79.8
Chronic conditions	75.2
Distance vision	74.6

Note. Selection frequency is the percentage of 500 household-bootstrap samples in which a predictor received a nonzero coefficient at the specified penalty. The 20 most frequently retained predictors are shown. Selection frequency measures stability under resampling and should not be interpreted as an effect size, causal importance, or incremental contribution to prediction. IADL = instrumental activities of daily living; CES-D = Center for Epidemiologic Studies Depression Scale.

Table S6

Composition of the Folds Used in Internal-External Validation

Fold	Provinces	n	Events	Remission rate (%)
1	Anhui, Guangdong, Heilongjiang, Henan, Jiangxi, Sichuan	1,468	297	20.2
2	Hebei, Jilin, Liaoning, Qinghai, Shanxi, Zhejiang	692	128	18.5
3	Guizhou, Hunan, Inner Mongolia, Shaanxi, Shandong	865	199	23.0
4	Beijing, Chongqing, Fujian, Jiangsu, Shanghai	272	55	20.2
5	Gansu, Guangxi, Hubei, Tianjin, Yunnan	820	139	17.0
**Total**	**27 provinces**	**4,117**	**818**	**19.9**

Note. Folds were used in the internal–external validation reported in Table 5 and Figure 7. Province was the grouping unit, and the 27 provinces represented in the analytic sample were allocated at random to five folds using a fixed random seed; the folds are therefore groups of provinces rather than administratively or economically defined regions. Provinces are identified from the first two digits of the CHARLS individual identifier, which correspond to the province codes given in the CHARLS primary sampling unit file. Xinjiang is represented in the CHARLS sampling frame but contributed no participants to the analytic sample after eligibility and follow-up criteria were applied. Fold sizes differ substantially, from 272 to 1,468 participants, so the held-out C-statistics are not equally precise and the spread across folds should be interpreted accordingly.

Figure S1

Iteration Trace Plots for Multiple Imputation by Chained Equations

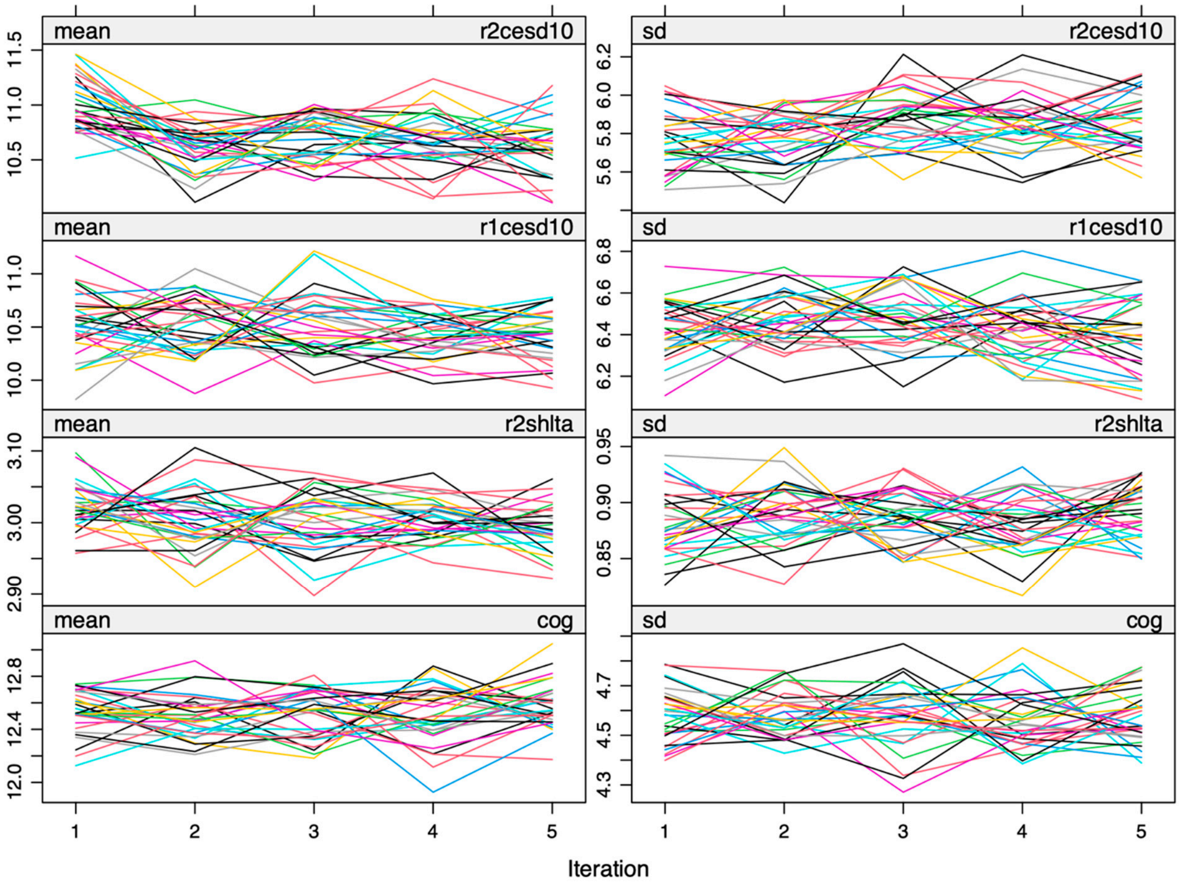

Note. Each panel plots the mean (left column) and standard deviation (right column) of the imputed values against iteration number, with one line per imputed dataset (m = 30, five iterations). Variables shown are the prior-wave depressive-severity measures (r2cesd10 = 2013 CES-D-10 score; r1cesd10 = 2011 CES-D-10 score), lagged self-rated health (r2shlta), and the global cognition composite (cog). These predictors were selected for display because they carried the largest penalized coefficients and among the highest proportions of missing data. Overlapping chains without separation into distinct bands, and the absence of systematic trend across iterations, indicate satisfactory convergence for these predictors. Among the full set of incomplete variables, some residual drift was observed for the two intergenerational-transfer variables, which had the highest proportions of missing data and received negligible penalized weights. Trace plots for all incomplete variables are available from the corresponding author on request. CES-D-10 = 10-item Center for Epidemiologic Studies Depression Scale.

TRIPOD+AI Checklist

Section / Topic	Item	Development / evaluation	Checklist item	Reported on page
**TITLE**				
Title	1	D;E	Identify the study as developing or evaluating the performance of a multivariable prediction model, the target population, and the outcome to be predicted.	p. 1 (title)
**ABSTRACT**				
Abstract	2	D;E	See TRIPOD+AI for Abstracts checklist.	p. 2 (Abstract)
**INTRODUCTION**				
Background	3a	D;E	Explain the healthcare context (including whether diagnostic or prognostic) and rationale for developing or evaluating the prediction model, including references to existing models.	pp. 3-4 (Introduction)
	3b	D;E	Describe the target population and the intended purpose of the prediction model in the context of the care pathway, including its intended users (e.g., healthcare professionals, patients, public).	p. 4 (Introduction)
	3c	D;E	Describe any known health inequalities between sociodemographic groups.	pp. 3 and 26-28 (Introduction and Discussion)
Objectives	4	D;E	Specify the study objectives, including whether the study describes the development or validation of a prediction model (or both).	p. 4 (Introduction, final paragraph)
**METHODS**				
Data	5a	D;E	Describe the sources of data separately for the development and	p. 4 (Study design and data source)

			evaluation datasets (e.g., randomised trial, cohort, routine care or registry data), the rationale for using these data, and representativeness of the data.	
	5b	D;E	Specify the dates of the collected participant data, including start and end of participant accrual; and, if applicable, end of follow-up.	pp. 4-5 (2011, 2013, 2015, 2018, and 2020 waves)
Participants	6a	D;E	Specify key elements of the study setting (e.g., primary care, secondary care, general population) including the number and location of centres.	p. 4 (Study design and data source)
	6b	D;E	Describe the eligibility criteria for study participants.	pp. 4-5 (Participants, prediction origin, and eligibility)
	6c	D;E	Give details of any treatments received, and how they were handled during model development or evaluation, if relevant.	pp. 6, 25, and 28 (treatment data not comprehensively available)
Data preparation	7	D;E	Describe any data pre-processing and quality checking, including whether this was similar across relevant sociodemographic groups.	pp. 6-7 (predictor processing, transformations, and imputation checks); no sociodemographic-group-specific quality checks reported
Outcome	8a	D;E	Clearly define the outcome that is being predicted and the time horizon, including how and when assessed, the rationale for choosing this outcome, and whether the method of outcome assessment is consistent across sociodemographic groups.	p. 5 (Outcome)
	8b	D;E	If outcome assessment requires subjective	Not applicable; the outcome was defined

			interpretation, describe the qualifications and demographic characteristics of the outcome assessors.	from participant-reported CES-D-10 scores (p. 5)
	8c	D;E	Report any actions to blind assessment of the outcome to be predicted.	Not reported; outcome assessments occurred after the prediction landmark (p. 5)
Predictors	9a	D	Describe the choice of initial predictors (e.g., literature, previous models, all available predictors) and any pre-selection of predictors before model building.	pp. 5-7 (Candidate predictors and Model development)
	9b	D;E	Clearly define all predictors, including how and when they were measured (and any actions to blind assessment of predictors for the outcome and other predictors).	pp. 5-6 and Supplementary Table S1
	9c	D;E	If predictor measurement requires subjective interpretation, describe the qualifications and demographic characteristics of the predictor assessors.	Not applicable; no predictor required assessor interpretation as defined in this item
Sample size	10	D;E	Explain how the study size was arrived at (separately for development and evaluation), and justify that the study size was sufficient to answer the research question. Include details of any sample size calculation.	p. 6 (Sample size)
Missing data	11	D;E	Describe how missing data were handled. Provide reasons for omitting any data.	p. 7 (Missing data)
Analytical methods	12a	D	Describe how the data were used (e.g., for development and evaluation of model	pp. 7-8 (Model development and Internal validation)

			performance) in the analysis, including whether the data were partitioned, considering any sample size requirements.	
	12b	D	Depending on the type of model, describe how predictors were handled in the analyses (functional form, rescaling, transformation, or any standardisation).	p. 6 (Candidate predictors)
	12c	D	Specify the type of model, rationale, all model-building steps, including any hyperparameter tuning, and method for internal validation.	p. 7 (Model development)
	12d	D;E	Describe if and how any heterogeneity in estimates of model parameter values and model performance was handled and quantified across clusters (e.g., hospitals, countries).	pp. 7-8 (household clustering and geographic internal-external validation)
	12e	D;E	Specify all measures and plots used (and their rationale) to evaluate model performance (e.g., discrimination, calibration, clinical utility) and, if relevant, to compare multiple models.	p. 8 (Internal validation and model performance)
	12f	E	Describe any model updating (e.g., recalibration) arising from the model evaluation, either overall or for particular sociodemographic groups or settings.	Not applicable; no model updating was performed
	12g	E	For model evaluation, describe how the model predictions were calculated (e.g., formula, code, object,	pp. 7-8 and Supplementary Table S3

			application programming interface).	
Class imbalance	13	D;E	If class imbalance methods were used, state why and how this was done, and any subsequent methods to recalibrate the model or the model predictions.	Not applicable; no class-imbalance method was used
Fairness	14	D;E	Describe any approaches that were used to address model fairness and their rationale.	No formal fairness method was used; future fairness evaluation is discussed on pp. 26 and 28
Model output	15	D	Specify the output of the prediction model (e.g., probabilities, classification). Provide details and rationale for any classification and how the thresholds were identified.	pp. 7-8 and 19-20 (probabilities and exploratory decision thresholds)
Training vs evaluation	16	D;E	Identify any differences between the development and evaluation data in healthcare setting, eligibility criteria, outcome, and predictors.	Not applicable; evaluation used internal and internal-external validation within CHARLS (p. 8)
Ethical approval	17	D;E	Name the institutional research board or ethics committee that approved the study and describe the participant-informed consent or the ethics committee waiver of informed consent.	p. 1 (Institutional Review Board and Informed Consent statements)
**OPEN SCIENCE**				
Funding	18a	D;E	Give the source of funding and the role of the funders for the present study.	p. 1 (Funding)
Conflicts of interest	18b	D;E	Declare any conflicts of interest and financial disclosures for all authors.	p. 1 (Conflicts of Interest)
Protocol	18c	D;E	Indicate where the study protocol can be accessed or state that a protocol was not prepared.	Not reported in the final manuscript

Registration	18d	D;E	Provide registration information for the study, including register name and registration number, or state that the study was not registered.	Not reported in the final manuscript
Data sharing	18e	D;E	Provide details of the availability of the study data.	p. 1 (Data Availability Statement)
Code sharing	18f	D;E	Provide details of the availability of the analytical code.	p. 1 (Data Availability Statement)
**PATIENT & PUBLIC INVOLVEMENT**				
Patient & public involvement	19	D;E	Provide details of any patient and public involvement during the design, conduct, reporting, interpretation, or dissemination of the study or state no involvement.	Not reported in the final manuscript
**RESULTS**				
Participants	20a	D;E	Describe the flow of participants through the study, including the number of participants with and without the outcome and, if applicable, a summary of the follow-up time. A diagram may be helpful.	pp. 9-10 (Participant flow and Figure 1)
	20b	D;E	Report the characteristics overall and, where applicable, for each data source or setting, including the key dates, key predictors, treatments received, sample size, number of outcome events, follow-up time, and amount of missing data. Report any differences across key demographic groups.	pp. 10-13 (Table 1); key study dates on pp. 4-5
	20c	E	For model evaluation, show a comparison with the development data of the	Not applicable; there was no independent evaluation dataset

			distribution of important predictors (demographics, predictors, and outcome).	
Model development	21	D;E	Specify the number of participants and outcome events in each analysis (e.g., for model development, hyperparameter tuning, model evaluation).	pp. 9, 17, and 22 (participant/event counts and analysis-specific sample sizes)
Model specification	22	D	Provide details of the full prediction model (e.g., formula, code, object) to allow predictions in new individuals and to enable third-party evaluation and implementation, including any restrictions to access or re-use.	Supplementary Table S3 (complete model specification); referenced on p. 8
Model performance	23a	D;E	Report model performance estimates with confidence intervals, including for any key subgroups (e.g., sociodemographic). Consider plots to aid presentation.	pp. 17-24 (Table 3, Figures 3-7, and internal-external validation results)
	23b	D;E	If examined, report results of any heterogeneity in model performance across clusters.	pp. 23-24 (geographic internal-external validation); no formal heterogeneity estimate was calculated
Model updating	24	E	Report the results from any model updating, including the updated model and subsequent performance.	Not applicable; no model updating was performed
**DISCUSSION**				
Interpretation	25	D;E	Give an overall interpretation of the main results, including issues of fairness in the context of the objectives and previous studies.	pp. 24-28 (Discussion and Conclusions)
Limitations	26	D;E	Discuss any limitations of the study (such as a non-representative sample, sample size, overfitting,	pp. 27-28 (Strengths and limitations)

			missing data) and their effects on any biases, statistical uncertainty, and generalizability.	
Usability	27a	D	Describe how poor quality or unavailable input data (e.g., predictor values) should be assessed and handled when implementing the prediction model.	Not reported; the model is not ready for implementation (pp. 25-28)
	27b	D	Specify whether users will be required to interact in the handling of the input data or use of the model, and what level of expertise is required of users.	Not reported; an end-user implementation tool was not developed
	27c	D;E	Discuss any next steps for future research, with a specific view to applicability and generalizability of the model.	pp. 27-28 (future validation, recalibration, fairness, and intervention research)

From: Collins GS, Moons KGM, Dhiman P, et al. BMJ 2024;385:e078378. doi:10.1136/bmj-2023-078378
